# Quaternized Polysulfones as Matrix for the Development of Broad-Spectrum Antimicrobial Coatings for Medical Devices

**DOI:** 10.3390/polym17131869

**Published:** 2025-07-03

**Authors:** Oana Dumbrava, Irina Rosca, Daniela Ailincai, Luminita Marin

**Affiliations:** “Petru Poni” Institute of Macromolecular Chemistry, 700487 Iasi, Romania; dumbrava.oana@icmpp.ro (O.D.); rosca.irina@icmpp.ro (I.R.)

**Keywords:** quaternized polysulfone, coatings, amphotericin B, norfloxacin, antimicrobial, antioxidant

## Abstract

The development and application of antimicrobial coatings has become increasingly important in both medical and industrial settings due to the rising threat of microbial contamination and antibiotic resistance. This paper focuses on the formulation, characterization, and investigation of coatings based on quaternized polysulfone, which are designed to encapsulate two broad-spectrum antimicrobial drugs with complementary activity, amphotericin B (AmB) and norfloxacin (NFX), with the primary aim of inhibiting pathogen colonization on surgical instruments. Structural characterization using FTIR, ^1^H-NMR, and UV-Vis spectroscopy, along with supramolecular analysis via X-ray diffraction and polarized optical microscopy (POM), revealed strong physical interactions between the drugs and the quaternized polysulfone matrix. Scanning electron microscopy (SEM) confirmed a uniform distribution of the antimicrobial agents within the polymeric matrix. Surface wettability, assessed through water contact angle measurements, indicated moderate hydrophilicity (70–90°). The coatings also exhibited notable antioxidant activity, showing a 12-fold increase in DPPH radical inhibition compared to the control. Furthermore, all formulations demonstrated strong antimicrobial efficacy against three reference strains frequently associated with hospital-acquired infections, *S. aureus*, *E. coli*, and *C. albicans*, with inhibition zones ranging from 32 to 39.67 mm for bacterial strains and 13.86 to 20.86 mm for *C. albicans*. These data points indicate that these materials may be useful as antimicrobial coatings.

## 1. Introduction

In recent decades, medical devices, especially those used to diagnose, monitor, and treat injuries or diseases, have made a remarkable contribution to prolonging and improving the lives of people around the world [1,2]. On the other hand, they constitute the main route of transmission for 50% of nosocomial infections, which endanger the health and, in some cases, the lives of patients [3,4,5]. As an example, there is a high incidence of infections during surgical procedures, particularly at the surgical site, which result in postoperative complications, the need for additional treatment, prolonged hospital stays, and even mortality [6,7]. Although surgical instruments are decontaminated and sterilized between surgeries to prevent cross-transmission, they can become contaminated during surgical procedures through contact with resident skin flora or microbes from the digestive tract, such as the stomach, duodenum, and colon. Thus, surgical instruments could act as a mean of spreading microorganisms into the surgical field [8].

Developing coatings that are easy to apply to existing medical devices and that can prevent bacterial adhesion and growth without harming the surrounding tissue could both minimize the occurrence of surgical device-associated infections and improve patient quality of life. Attempts to do so have revealed that coatings which offer controlled release of active substances, such as antimicrobial agents, effectively prevent infections associated with medical devices [9,10,11]. Many synthetic polymers, such as poly(etheretherketone), poly(terpinol), poly(imide), poly(amide), poly(ester), poly(dimethylsiloxane), poly(acrylic acid), polyacrylamide, poly(ethylen imine), poly(ethylenglycol), polypyrrole, poly(butyl acrylatemethyl methacrylate), poly (diallyldimethylammonium chloride), poly (methacrylic acid), poly(tetrafluorethylene), poly(allylamine hydrochloride), poly(3,4-ethylenedioxythiophene), and various combinations thereof have been used as matrices to design various drug delivery coatings, with promising results [12,13]. Nevertheless, none of these coatings have reached the market, leaving room for further research and developments.

Polysulfone (PSF) is a high-performance polymer recognized for its high thermal stability, mechanical strength, and chemical resistance. These properties, along with its inherent transparency and biocompatibility, make it an ideal material in biomedical devices, particularly those exposed to harsh sterilization processes, such as steam autoclaving or chemical disinfectants. Furthermore, the chemical modification of PSF with permanent positive charge by quaternization significantly improves its performance for biomedical application, such as enhanced antimicrobial activity, improved hemocompatibility and hydrophilicity, and reduced protein fouling, all of which are critical for reducing immune responses and biofouling in blood-contacting devices [14,15,16]. These advantages make quaternary polysulfone particularly appealing as a coating for various medical devices. However, while PSF has been explored as a coating for various medical devices, to the best of our knowledge, quaternary polysulfone has not been investigated in this context [17,18,19].

A review of the literature indicates that most efforts in the field of medical device coatings have focused on achieving prolonged antibacterial activity, while the prevention and control of fungal infections, particularly those caused by *Candida* species, have received relatively less attention [20,21]. This represents a significant gap, given that candidemia continues to pose a major clinical challenge with high mortality rates [22,23,24]. Moreover, the treatment of such infections is complicated by the limited effectiveness of intravenous antifungal agents like amphotericin B (AmB) against biofilms, as well as the severe side effects associated with its use, which often limit it to a last-resort therapy [25].

Therefore, given the current state of the research, this paper aimed to develop coatings that not only inhibit the growth of bacteria and fungi, but also actively eliminate these microorganisms. With this goal in mind, the present study explores the use of quaternized polysulfone as a matrix for the encapsulation of two potent antimicrobial agents: norfloxacin (NFX), a broad-spectrum quinolone antibiotic, and amphotericin B (AmB), a polyene antifungal drug. This approach aims to create coatings with enhanced antibacterial and antifungal properties, specifically designed to prevent biofilm formation on medical device surfaces. The coatings were designed with a rational, multi-level strategy: (i) ability of quinolones to enhance the efficacy of AmB through synergistic effects, as prior demonstrated in some studies [26]; (ii) the potential of the chemical structures of both the polymer and the drugs to promote strong electrostatic interactions, which can support sustained drug release; (iii) the complementary bioactivities of the drugs, with the combination of NFX providing antibacterial effects and AmB offering antifungal action resulting in coatings with broad-spectrum antimicrobial properties; and (iv) the inherent antimicrobial activity of the quaternized polysulfone matrix, with the potential to effectively inhibit a wide range of pathogenic strains, thereby enhancing the overall antimicrobial efficacy of the coating.

It is important to note that this coating design is novel, marking the first use of quaternized polysulfone as a matrix for developing antimicrobial coatings.

## 2. Materials and Methods

### 2.1. Materials

Polysulfone (M_w_ = 35,000 Da and M_n_~16,000 Da) was purchased from Sigma Aldrich (Darmstadt, Germany) and purified before being subjected to chemical modification by dissolution in chloroform and precipitation in methanol. Subsequently, the polymer was oven-dried for 72 h at 40 °C. Tin tetrachloride, trimethylchlorosilane, N,N-dimethylformamide (DMF), N,N-dimethylsulfoneamide (DMSO), norfloxacin (NFX, 98%), 2,2-Diphenyl-1-picrylhydrazyl (DPPH), and acetic acid were purchased from Sigma Aldrich (Darmstadt, Germany) and were used as received. Chloroform, methanol, and acetone were purchased from Chemical Company (Iasi, Romania) and distilled before use. N,N-dimethylbutylamine was purchased from TCI Chemicals (Tokyo, Japan). Amphotericin B (AmB, 85%) was provided by Thermo Scientific Chemicals (Wien, Austria).

### 2.2. Synthesis of QPSF

QPSF was obtained by reacting chloromethylated polysulfone (CMPSF) with N,N-dimethylbutylamine according to a procedure previously described in the literature [27]. Based on the ^1^H-NMR spectra, the degree of substitution of the functionalized polysulfone was 1.4. The detailed steps involved in the synthesis pathway of QPSF, as well as its structural characterization, are presented in the supporting material (Figures S2–S6).

### 2.3. Preparation of Formulations

**Figure 1:**
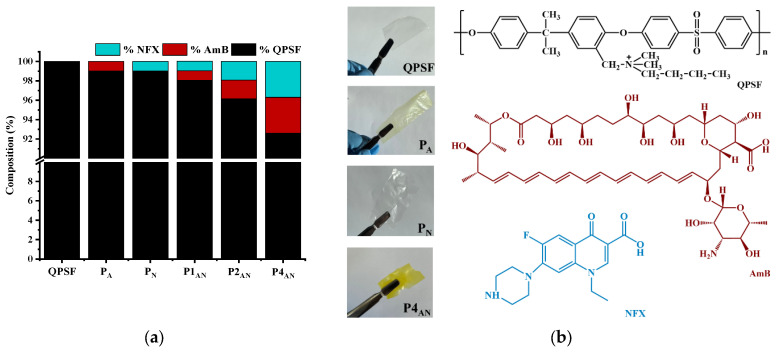
(**a**) The composition of the prepared coatings and their codes; (**b**) chemical structures of the three components used in this study and pictures of representative samples.

A series of five coatings containing AmB and/or NFX were obtained by varying the mass ratio between QPSF and the two drugs. QPSF was initially dissolved in DMF to form homogeneous 25% solutions (*w*/*v*). Separately, AmB, NFX, and their combined mixtures were solubilized in DMSO and acetic acid, sonicated for 30 min at 50 °C, and subsequently added dropwise to the QPSF solution under vigorous stirring (1000 rpm). The final concentration of the polymer-drug mixtures was adjusted to 20% (*w*/*v*), with the drug being set at 1%, 2%, and 4% (*w*/*w*) relative to the QPSF matrix (Figure 1). To ensure uniform dispersion, the mixtures were vortexed for 5 min, followed by 30 min of magnetic stirring, a sequence repeated three times. After thorough mixing and the removal of air bubbles, the resulting formulations were cast onto Teflon sheets and dried at 70 °C for 48 h to obtain defect-free coatings. A QPSF sample was prepared using the same procedure as a reference for comparison. The sample codes and their composition are provided in Figure 1. This preparation procedure resulted in complete (100%) encapsulation efficiency of the drugs within the polymeric matrix, a value that was subsequently considered in all further investigations.

### 2.4. Characterization

**Scheme 1:**
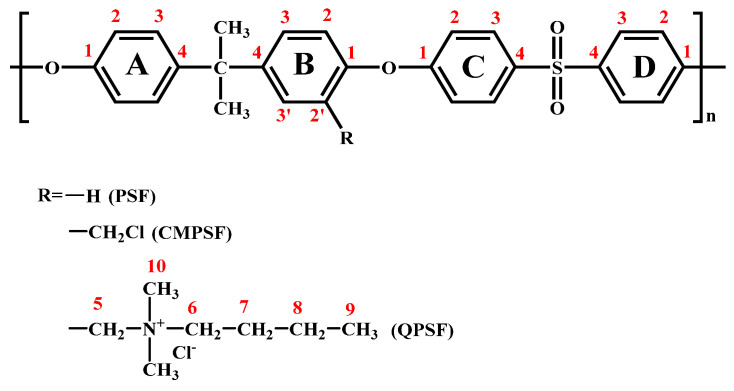
Atoms numbering for NMR assignments.

The NMR spectra were recorded with a Bruker Avance NEO 400 MHz spectrometer (Bruker BioSpin, Ettlingen, Germany). The samples were dissolved in deuterated dimethylsulfoxide (DMSO-d_6_), and their spectra were calibrated on the solvent residual peak (2.512 ppm). Atom numbering for NMR assignments is shown in the Scheme 1. 

In order to assess the intermolecular interactions between the QPSF matrix and the drugs, spectra at temperatures from 21 to 50 °C were recorded. To establish the statistical significance of the shifting of the signals, the spectra were recorded on five different specimens of each sample.

Fourier-Transform infrared (FTIR) spectra of the PSF, CMPSF, and QPSF polymers, as well as the spectra of the films based on QPSF, AmB, and/or NFX were recorded with a FTIR Bruker Vertex 70 Spectrophotometer (Bruker, Ettligen, Germany), from 4000 to 600 cm^−1^, using the ATR method. The overlapped bands from the 3700–3100 cm^−1^ region in the FTIR spectrum of the investigated samples were highlighted with the second derivative analysis of the spectra. These bands were then deconvoluted with a curve-fitting assay, and their areas calculated using a 50% Lorentzian and 50% Gaussian function. The obtained spectra were processed with OPUS 6.5 software.

UV-Vis spectra of QPSF and the five formulations were registered in the solid state with an Agilent Cary 60 UV-Vis spectrophotometer (Agilent Technologies, Inc. Headquarters, Santa Clara, CA, USA).

The nature of the P_A_, P_N_, P1_AN_, P2_AN_, and P4_AN_ coatings and the AmB, NFX, and QPSF reference samples was studied by recording the wide-angle X-ray diffraction with a Rigaku Miniflex 600 diffractometer (Rigaku, Tokyo, Japan) with CuKα-emission, at room temperature, from 5 to 50° (2θ), a scanning step of 0.06°, and a recording speed of 4°/min. Polarized optical microscopy (POM) with a Zeiss Axio Imager M2 microscope (Carl Zeiss Microscopy GmbH, Oberkochen, Germany) was also conducted.

The topography and cross-section morphology, as well as the elemental mapping of the understudied materials, were investigated using a scanning electron microscope SEM EDAX—Quanta 200 (FEI Company, Hillsboro, OR, USA).

Static contact angle values were determined with the aid of a KSV CAM-101 goniometer (KSV Instruments, Helsinki, Finland), equipped with a Hamilton syringe and a CDD camera, by placing a drop of 1 μL water (W), ethylene glycol (EG), or diiodomethane (DIM) on the surface of the investigated materials. The measurements were performed three times for each test liquid, and the average values were reported.

The radical scavenging activity (RSA) of the samples in the solid state was assayed using the DPPH method [28]. Pieces of films weighing 10 mg each were introduced in 1.5 mL of methanolic solution of DPPH (0.025 mg/mL). After 1 h of incubation at 37 °C in the dark, the UV-Vis absorbance of the supernatant was registered at 517 nm, and the RSA for each sample was calculated by using the following equation:

(1) $$RSA=\frac{A_{DPPH}-A_{s}}{A_{DPPH}}\cdot 100$$

where $A_{DPPH}$ is the absorbance of the DPPH solution and $A_{s}$ is the absorption of DPPH solution after one hour of exposure to each sample. To analyse the results, three controls were used: (i) ascorbic acid, a positive control, at the same concentration as the samples; (ii) norfloxacin and (iii) amphotericin B—the same drug concentration as in the P4_AN_ sample.

The drug release was evaluated using a protocol designed to simulate topical drug delivery conditions [29]. First, 6 × 6 mm pieces of films, each weighing 2.7 ± 1 mg, were placed onto 10 × 10 mm filter papers pre-wetted with 50 μL buffer solution at room temperature. The filter paper was replaced at specific intervals, and the drug from the removed paper was extracted using a 2 mL buffer solution, then subjected to UV-Vis measurement to determine the specific absorbance. The drug concentration released was calculated by fitting the obtained values of absorbance on a prior drawn calibration curve (Figure S17). The cumulative release of drug from the samples was determined by applying the Equation (2):

(2) $$\%\;drug=\frac{10C_{n}-2\sum C_{n-1}}{m_{0}}\cdot 100$$

where $C_{n}$ and $C_{n-1}$ C_n−1_ represent the concentrations of the drug in the supernatant after n and n−1 n − 1 withdrawing steps, respectively, and $m_{0}$ is the drug amount in the initial samples (1.27 × 10^−5^–11.19 × 10^−5^ NFX and 1.31 × 10^−5^–13.12 × 10^−5^ g AmB).

The in vitro release mechanisms were investigated by fitting the experimental release data on the following mathematical models:

(i) Zero order model: $Q_{t}=K_{0}\cdot t$, where $Q_{t}$ is the amount of drug dissolved in the time t and $K_{0}$ is the Zero order release constant.
(ii) First order model: $logQ_{t}=logQ_{0}+K\cdot t/2.303$, where $Q_{t}$ is the amount of drug released in the time t, $Q_{0}$ is the initial amount of drug and K is the first order release constant.
(iii) Higuchi model: $Q_{t}=K_{H}\cdot t^{\frac{1}{2}}$, where $Q_{t}$ is the amount of drug released in the time t and $K_{H}$ is the Higuchi dissolution constant.
(iv) Hixson–Crowell model: $W_{0}^{\frac{1}{3}}-W_{1}^{\frac{1}{3}}=K\cdot t$, where $W_{0}$ is the initial amount of drug in the coating, $W_{t}$ is the remaining amount of drug in the formulation at time t and K is a constant.
(v) Korsmeyer–Peppas model: $\frac{M_{t}}{M_{\infty }}=K\cdot t^{n}$, where $\frac{M_{t}}{M_{\infty }}$ is the fraction of drug released at the time t, K is the release rate constant and n is the release exponent.

The antimicrobial activity of samples was evaluated against three different reference strains *Staphylococcus aureus* ATCC25923 (*S. aureus*), *Escherichia coli* ATCC25922 (*E. coli*), and *Candida albicans* ATCC90028 (*C. albicans*) using two different methods: a modified Japanese industrial standard JIS Z2801:2000 [30] and the disk diffusion assay method [31]. All microorganisms were stored at −80 °C in 20% glycerol. The bacterial and yeast strains were refreshed for 24 h at 37 °C in nutrient agar (NA) and Sabouraud dextrose agar (SDA), respectively.

In the case of the Japanese industrial standard, the antimicrobial activity of the samples was assessed by OD600, a common method for estimating bacterial concentration, and MTS assay (CellTiter 96^®^ AQueous One Solution Cell Proliferation Assay—Promega, Madison, WI, USA). Samples of 1 cm^2^ were placed in a sterile Petri dish, and 0.1 mL of the inoculum (0.5 McFarland standard) was instilled on the sample surface and left to incubate for 24 h at 37 °C. After incubation, the samples were rinsed repeatedly, and the solution removed from the rinsed samples were plated in 96-well plates in a final volume of 0.1 mL Nutrient Broth or Sabouraud Dextrose Broth/well and incubated. Also, microbial control samples (0.5 McFarland same concentration as the tested inoculum was used on the tested samples) were incubated in the same conditions. In the case of MTS assay, the samples were removed from the plates after 23 h, and 20 µL of MTS reagent was added to them for 1 h. After the formazan formation, the absorbance recording was performed at 490 nm on a FLUOstar^®^ Omega microplate reader (BMG LABTECH, Ortenberg, Germany). Experiments were performed in triplicate, and treated cell viability was expressed as percentage of control cells’ viability. Graphical data were expressed as means ± standard error of the mean. For the observation of *Candida* cells on the materials’ surfaces, samples from the culture medium were collected, sterilized for 15 min under a UV lamp, and then subjected to SEM analysis of their surfaces.

According to the protocol of disk diffusion assay, 0.1 mL from each inoculum were spread onto NA/SDA plates, and samples of 10 mm and 7.5 mg were added. To evaluate the antimicrobial properties, the growth inhibition was measured under standard conditions after 24 h of incubation at 37 °C. All tests were carried out in triplicate to verify the results. After incubation, the samples were analyzed with SCAN1200^®^, version 8.6.10.0 (Interscience, Saint-Nom-la-Bretèche, France), and were expressed as the mean ± standard deviation (SD).

The obtained results for the contact angle, antioxidant and bacteriostatic activity experiments, as well as the chemical shift values of the methylene bridges, were expressed as the arithmetic mean ± the standard deviation (S.D.) of the average of the values for each determined parameter. They were statistically analyzed using GraphPad Prism 9.0 software by one way or two-way ANOVA method. The results were considered statistically significant when *p* < 0.05.

## 3. Results

A series of five formulations was developed by combining quaternized polysulfone with the antifungal drug AmB and the broad-spectrum antibiotic NFX, with the aim of creating bioactive coatings for biomedical devices. The rational design behind these coatings leveraged the complementary antimicrobial profiles of both AmB and NFX, which together provide both antifungal and antibacterial activity. Additionally, the quaternary ammonium groups of QPSF are expected to interact with the carboxyl groups of both AmB and NFX via electrostatic forces, effectively anchoring the drugs within the polymer matrix. These electrostatic interactions, together with the extensive intermolecular forces arising from the complementary polar groups present in the polymer matrix (quaternary ammonium and electron-withdrawing sulfone groups) and in the two drugs (hydroxyl, carboxyl, and amine groups in AmB; carboxyl, carbonyl, fluorine, and secondary amine groups in NFX), are expected to enable a sustained drug release, thereby enhancing the long-term antimicrobial performance of the coatings (Figure 1).

### 3.1. Structural Characterization

Drug encapsulation within the quaternized polysulfone matrix was achieved by solution mixing followed by casting, resulting in maximum encapsulation efficiency. The qualitative encapsulation of drugs into the QPSF matrix was investigated by ^1^H-NMR, FTIR, and UV-Vis spectroscopy.

**Figure 2:**
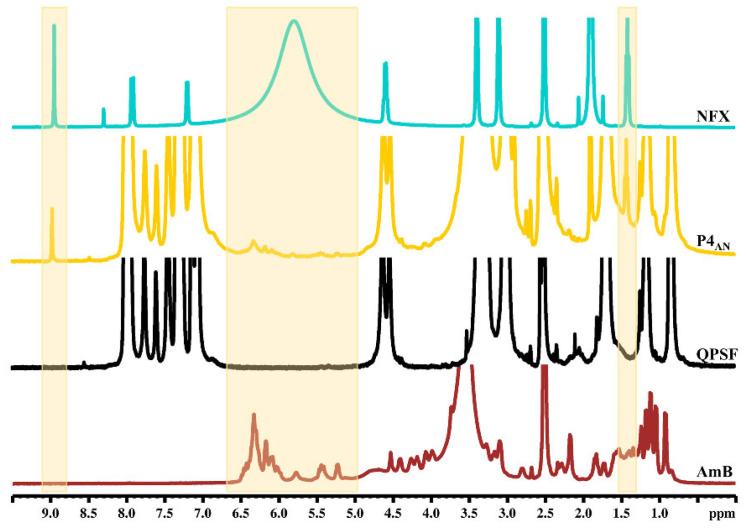
^1^H-NMR spectra of P4_AN_ coating with its components, NFX, AmB, and QPSF.

The ^1^H-NMR spectra of the coatings were recorded and interpreted in comparison with the spectra of the neat components. The ^1^H-NMR spectrum of NFX (Figure 2) exhibited one singlet and two doublets in the aromatic region at 8.95, 7.95–7.9, and 7.2–7.18 ppm, each corresponding to one aromatic proton from the fluoroquinolone moiety, namely: the aromatic proton adjacent to the quinolone nitrogen atom and the aromatic protons in *ortho* and *meta* positions to the fluorine atom. The protons from the ethyl group appeared in the ^1^H-NMR spectrum as a multiplet and a triplet at 4.60–4.58 and 1.43–1.39 ppm, while the ones from the piperazine moiety appear at 3.9 and 3.1 ppm, corresponding to eight equivalent protons [32]. The ^1^H-NMR spectrum of AmB (Figure 2) exhibited characteristic signals located at 6.16–5.9 ppm and 5.46–5.4 ppm, which correspond to the olefinic protons from AmB structure. The peak around 5.23–5.22 ppm is assigned to the proton neighboring the ester group, while the protons in the *ortho* position relative to the ether group appeared around 4.5–4.4 ppm and 4.18 ppm. The protons located near the hydroxyl group appear in the region 4.2–3 ppm, the ones adjacent to the –CH_3_ group are present in the ^1^H-NMR spectrum at 2.33–2.27 and 1.73–1.71 ppm, and the protons near the –COOH functional group appear at 1.85–1.82 ppm. The methylene protons within the AmB structure were present in the 1.5–1.2 ppm domain, and the ones from the methyl group appeared as four doublets at 1.23–1.03 ppm [33].

The ^1^H-NMR spectrum of QPSF revealed the peaks characteristic of aromatic protons in the quaternized polysulfone structure, located at 7.85 and 7.00 ppm (protons of the unsubstituted phenylsulfone rings C and D), 7.70 and 7.63 ppm (monosubstituted H3′B and disubstituted H3′AB), 7.44 ppm (H3B), and 7.03–7.12 ppm (H2B and H2′B protons overlapped with H2A), as well as the signals characteristic of protons in the dimethyl butyl residue at 3.28, 3.03–2.99, 1.18–1.13, 1.67 (overlapped with the signal for –C(CH_3_)_2_ in the bisphenol unit), and 0.82 ppm (Figures S2–S6).

Despite the overlapping signals, the ^1^H-NMR spectra of the coatings showed distinct signals for both the QPSF matrix and the drugs. Thus, all the signals characteristic to QPSF were present in the spectra along with the singlet at 8.95 ppm and the multiplet at 1.44–1.41 ppm, characteristic of NFX, and the signals at 5.46–5.39 ppm and 5.21 ppm, characteristic of AmB.

**Figure 3:**
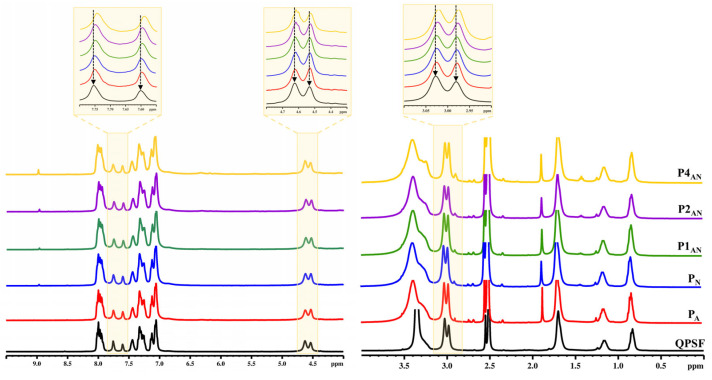
Comparative ^1^H-NMR spectra of QPSF and drug containing coatings.

A more detailed analysis of the ^1^H-NMR spectra of samples P_A_, P_N_, P1_AN_, P2_AN_, and P4_AN_, compared to the reference sample QPSF, revealed subtle but noteworthy changes, consistent with the occurrence of electrostatic interactions between the quaternary ammonium groups of the polymer and the carboxyl groups of the incorporated drugs (Figure 3). Specifically, the signals corresponding to the methylene protons adjacent to the quaternary nitrogen (H5 in Scheme 1) exhibited a gradual downfield shift with increasing drug concentration, shifting from 4.613–4.523 ppm in QPSF to around 4.608–4.521 ppm in P4_AN_. This shift modification was statistically significant for P2_AN_ and P4_AN_ (Figures S7 and S8). This trend suggests an increased likelihood of interactions between the polymer matrix and the encapsulated drugs. Although these shifts are statistically significant, it is important to note that the ^1^H-NMR spectra were recorded in highly dilute solutions, where Brownian motion reduces the extent of intermolecular interactions. This was proven by an ^1^H-NMR experiment recording the spectra at temperatures from 21 to 50 °C, which clearly showed the complementary shifting of the protons from QPSF and drugs. Thus, on one hand, the protons adjacent to the quaternary nitrogen shifted upfield (toward lower ppm values) as the temperature decreased, suggesting increased shielding that was likely due to attenuation of the nitrogen’s permanent positive charge through intermolecular interactions. On the other hand, the protons near electron-rich groups, such as carboxyl and nitrogen, showed downfield shifts (toward higher ppm values), indicating deshielding effects consistent with enhanced electron-withdrawing behavior at lower temperatures (Figure S9). This experiment strongly supports the existence of electrostatic interactions between quaternary nitrogen of QPSF and electron-rich groups of the drugs.

**Figure 4:**
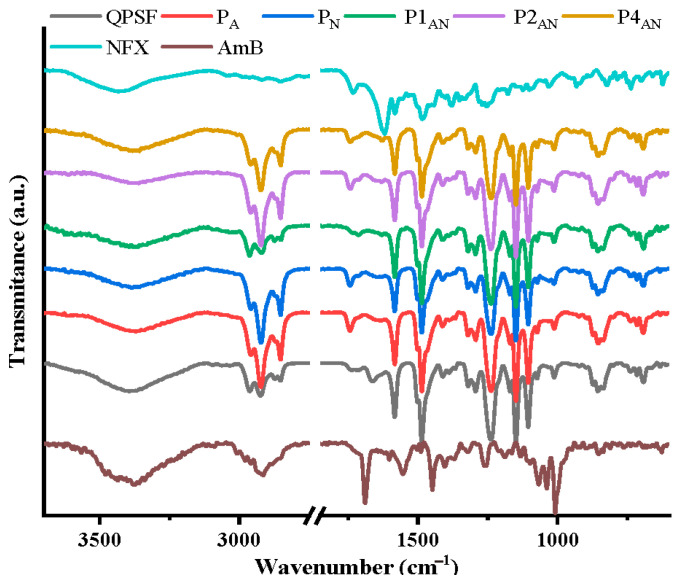
FTIR spectra of the investigated coatings, as well as of AmB and NFX drugs.

FTIR spectroscopy was used in order to confirm the presence of the components into coatings, as well as to assess the potential interactions between them. To this aim, the FTIR spectra of the coatings were analysed and discussed in comparison with the spectra of neat components, QPSF, AmB and NFX drugs (Figure 4). QPSF presented absorption bands corresponding to the polysulfone chain among which the most notably are: aromatic stretching at 1580 and 1490 cm^−1^, the C–O–C stretching vibrations at 1239 cm^−1^, O–S–O asymmetric stretching at 1320 cm^−1^ and O–S–O symmetric stretching at 1150 cm^−1^, and a broad band with a maximum at 3395 cm^−1^ characteristic of the hydrogen bonds which may form between the quaternary nitrogen and the atmospheric water due to its hydrophilicity (Figure S10) [34]. In the FTIR spectrum of NFX, a characteristic broad absorption band with a maximum at 3434 cm^−1^ appeared, which corresponds to the superposition of the OH vibration, intermolecular hydrogen bonds, and NH stretching vibrations of the amino fragment from the piperazinyl group. The absorption bands which appeared around 1732 cm^−1^ and around 1650–1600 cm^−1^ were assigned to the carbonyl C=O stretching vibration and the N-H bending vibration of the quinolone moiety, respectively. Furthermore, a characteristic absorption band of the NFX structure appeared at 1029 cm^−1^, which is associated with the C–F bond vibration [35]. In the case of AmB, absorption bands around 3420, 2926, 1692, 1573, and 1070 cm^−1^ were observed, which are specific to the -OH and -NH- stretching vibration, methylene stretching, C=O stretching of the carboxylic acid group, the polyene C=C stretching, and C–O stretching [36], in accordance with its chemical structure.

Comparative analysis of the FTIR spectra obtained for the drug containing formulations alongside their reference components (the polymer matrix and the individual drugs) revealed slight but consistent variations in both peak positions and intensities. Notable, the broad absorption occurred at 3395 cm^−1^ in the spectrum of QPSF, shifted progressively towards lower wavenumber as the drug content increased. Therefore, this band shifted to 3390 cm^−1^ in P_A_ and 3382 cm^−1^ in P_N_, while for the coatings containing both drugs, the absorption maximum progressively shifted further, to 3375 cm^−1^ in P4_AN_. This trend suggests the formation of strong intermolecular interactions between the polymer matrix and the encapsulated drugs. Deconvolution of the 3700–3100 cm^−1^ spectral region revealed three distinct bands in QPSF, increasing to five in the coatings containing a single drug (either AmB or NFX) and to eight in those incorporating both drugs (Figure S11). This trend reflects a growing diversity of interaction types, consistent with the chemical structures of the incorporated compounds. Given that QPSF features positively charged nitrogen atoms and electron-rich sulfone (SO_2_) groups, while AmB contains carboxyl, hydroxyl, and nitrogen functionalities, and NFX includes hydroxyl, nitrogen, and fluorine groups, the observed bands are plausibly attributed to a combination of hydrogen bonding and electrostatic interactions.

Therefore, both the NMR and FTIR spectra of the samples indicate that strong interactions occur between the polysulfone matrix and the encapsulated drugs, providing the necessary prerequisites for the development of sustained-release drug delivery systems.

**Figure 5:**
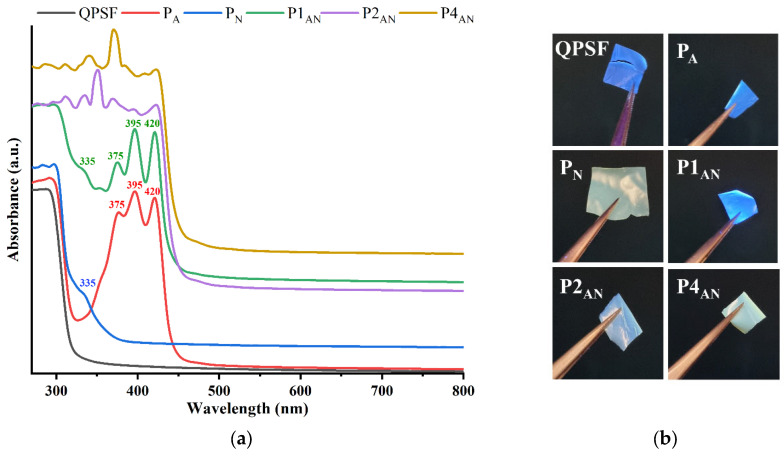
UV-Vis spectra of the obtained films (**a**) and images with the formulations under an UV-lamp (λ = 325 nm) (**b**).

**Figure 6:**
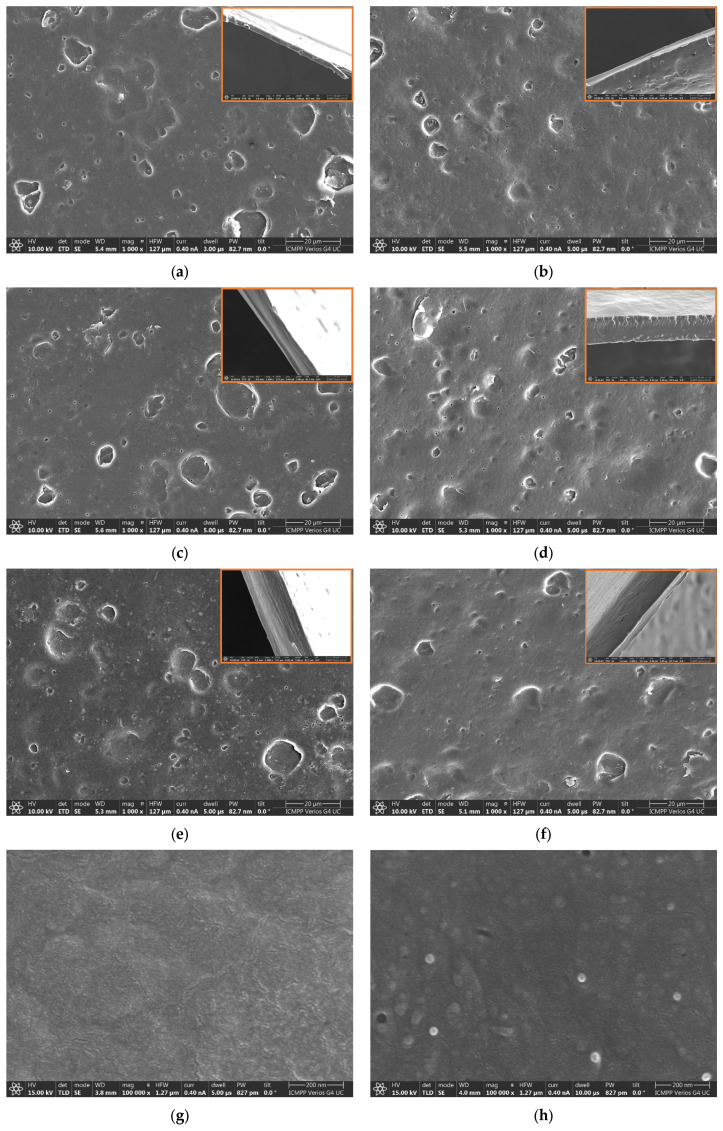
Surface and cross-section micrographs of (**a**,**g**) QPSF, (**b**) P_A_, (**c**) P_N_, (**d**) P1_AN_, (**e**) P2_AN_, and (**f**,**h**) P4_AN._

A complementary confirmation of the presence and interactions of the components in the coatings was provided by UV-Vis spectroscopy. Polysulfone exhibited characteristic absorption bands in the UV region, primarily below 300 nm, attributable to π-π* transitions within the aromatic rings and sulfone groups of its backbone (Figure 5a). The sample containing AmB (P_A_) showed an absorption profile reflecting the presence of AmB, with the appearance in the UV-Vis spectrum of three intense absorption maxima at 375, 395, and 420 nm, and a shoulder around 350 nm [37,38]. These bands are bathochromic shifted when compared to the positions of aggregated AmB (347, 365, 384, and 406 nm) and close to those reported for AmB solution (350, 368, 388, and 412 nm) (Figure S12). Furthermore, the band at 350 nm, whose intensity is considered an indicator for the aggregation of its molecules, has a very low intensity, as a shoulder [37] in accordance with very slight aggregation of the drug (Figure 6g,h; Figure S13). These suggest a fine dispersion of AmB into the polymeric matrix, similar to diluted solutions. Furthermore, the position of the bands is even more bathochromic, shifted by about 5 nm, in line with an extended electronic conjugation that decreased the HOMO-LUMO gap and, consequently, shifted the absorption band to lower energy. This is rationally expected to occur by interactions of the polymeric matrix with the drug, strengthening the conjugation of the polyene system. The sample P_N_, incorporating NFX, displayed a shoulder at 335 nm, aligning with the literature values for this drug [39]. The UV-Vis spectra of the samples containing both drugs (P1_AN_, P2_AN_ and P4_AN_) confirmed the presence of the two antimicrobial agents in the structure of the materials by the appearance of all their characteristic bands. The P1_AN_ spectrum showed that all bands had almost similar intensity, suggesting a fine dispersion of the drugs into matrix. Furthermore, samples P2_AN_ and P4_AN_, characterized by higher drug loadings compared to P1_AN_, displayed shifts in the positions of the absorption bands and variations in their number. Thus, the intensity of the band around 350 nm of AmB in P2_AN_ increased while the intensity of the other bands decreased, indicating its aggregation [37]. In the case of sample P4_AN_, the increase in the intensity of the 350 nm band was less obvious, and the one at 375 nm was the highest.

Remarkably, the characteristic QPSF absorption band shifted bathochromic in line with an increase in conjugation, which can be attributed to intermolecular interactions with the drugs, most likely π-π interactions, as well as interactions of the electron deficient sites of the drugs with the aromatic rings of the polymer. These complex changes in the absorption patterns depending on the amounts of drugs suggest the existence of complex interactions networks between the QPSF matrix and drugs as well as between drugs, most likely through the formation of dimers via carboxyl units.

The differences in absorbance were also reflected in variations in the color and intensity of the samples’ luminescence when exposed to UV illumination, further supporting the formation of new interactions among the components of the formulations, including interactions between the drug molecules (Figure 5b).

Additional information regarding the successful encapsulation of AmB and/or NFX drugs within the quaternized polysulfone matrix was derived from the EDAX mapping recorded for the investigated materials (Figure S14, Table S1). These data revealed the characteristic signal of the fluorine atom, specific to NFX, in samples P_N_, P1_AN_, P2_AN_, and P4_AN_, and its uniform distribution, confirming its homogeneous dispersion throughout the QPSF matrix. Furthermore, a reduction in the relative percentage of sulfur and chlorine atoms, observed with increasing drug content, is in accordance with the corresponding decrease in the dominance of polymer matrix.

### 3.2. Morphological Characterization

To investigate the morphology and distribution of the drugs into the polymeric matrix, surface and cross-sectional SEM images of the formulations were recorded (Figure 6 and Figure S15).

P_A_, P_N_, P1_AN_, P2_AN_, and P4_AN_ coatings displayed surface protuberances of approximately 3 μm, similar to morphologies typically observed in composites formed by dispersing low-molecular-weight compounds in a polymer matrix [40]. Similar protuberances were also observed in the neat QPSF film, suggesting that their formation is more likely driven by the presence of permanent positive charges promoting chain depletion, rather than by drug depot formation. This interpretation is supported by cross-sectional images, which revealed a compact internal morphology with no visible phase-separated domains. Nevertheless, a deeper view of the samples, using 50,000–100,000 magnification, revealed rare, small aggregates of 30–50 nm; their presence in the case of drug-containing sample and their absence in the case of QPSF, suggests the formation of some drug aggregates, as shown by UV-Vis.

**Figure 7:**
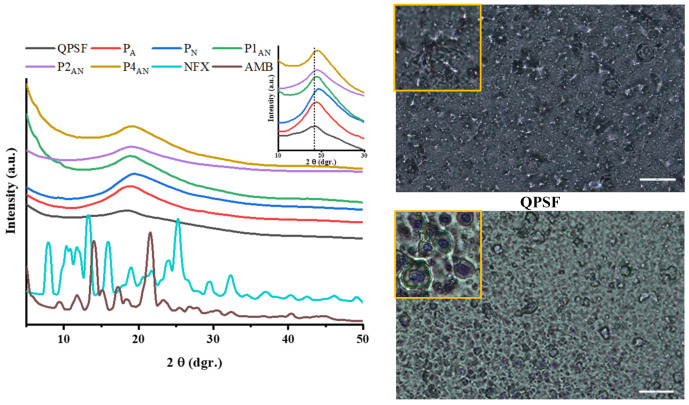

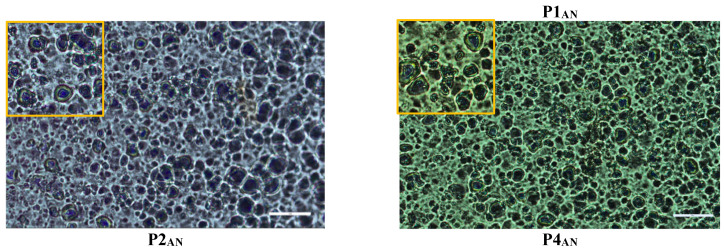
XRD diffractograms of the investigated coatings, AmB and NFX references and POM images of pristine QPSF film, as well as of the films containing both drugs—P1_AN_, P2_AN_, and P4_AN_ (scale: 50 µm).

Wide-angle X-ray diffraction (WAXD) and polarized optical microscopy (POM) are powerful techniques for characterizing drug delivery systems, providing valuable insights about the drug distribution within the polymer matrix, its uniformity, and its physical state. Figure 7 exhibits the XRD profiles of QPSF, AmB, NFX, and the five drug containing materials.

The XRD pattern of the QPSF sample displayed a broad, low-intensity reflection between 15° and 22° 2θ, peaking at 18.7° 2θ, which corresponds to a d-spacing of 4.8 Å. This suggests an overall amorphous structure with locally ordered domains, likely resulting from π–π interactions between certain polymer segments. The presence of these organized clusters is further supported by the observation of birefringent domains under polarized light (Figure 7) [41]. As expected, the diffractograms of the plain drugs displayed multiple diffraction peaks, in line with their high crystallinity [42,43].

The diffractograms of the formulations closely resembled that of QPSF, with no distinct diffraction peaks attributable to AmB or NFX. This suggests that the drugs were finely dispersed within the polymer matrix, likely due to intermolecular interactions with QPSF that inhibit crystallization, at least to the extent detectable by this technique [44]. However, a slight shift of the diffraction maxima toward higher angles and, consequently, a lower d-spacing was observed, suggesting that the presence of the drugs promoted coalescence of the polymeric chains into more compact ordered domains. This effect was further supported by the appearance of more pronounced birefringent domains under polarized light [40,45,46]. The lack of clear diffraction bands characteristic of the crystalline drugs is in agreement with rare aggregates observed in SEM, which, combined with the drug aggregation evidenced by UV-Vis, reinforces the idea of very fine drug pockets dispersed into the polymeric matrix.

### 3.3. Surface Properties

As the coating surface may interact directly with tissues and biological fluids, its characteristics, such as wettability and surface energy, were analyzed in depth in this study. In the case of blood-contacting devices and tissue engineering, a balanced hydrophilic/hydrophobic surface is essential [47]. While hydrophilic coatings provide superior lubricity, preventing friction with mucosa, which causes pain to the patient, they can compromise device handling due to their slippery nature. Hydrophobic coatings provide better grip, improving user control, but are less effective lubricants [48]. Depending on the application, a more hydrophilic or more hydrophobic surface may be preferred. However, for many devices that come into contact with living tissues, a surface with moderate wettability, neither highly hydrophilic nor highly hydrophobic, is often sufficient and desirable [49,50]. Furthermore, the identification of the surface characteristics can allow the chemistry of surface phenomena to be understood and also provide a first glimpse into their potential application.

**Figure 8:**
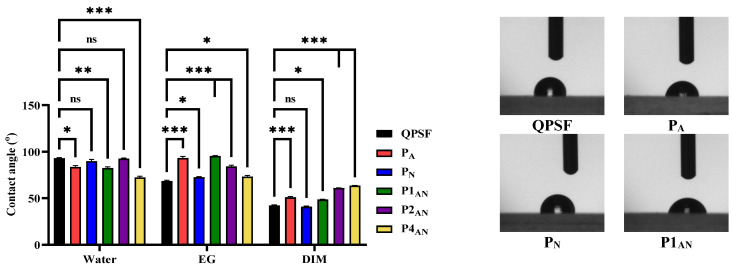
Contact angle of the investigated materials with water, ethylene glycol, and diiodomethane (* *p* < 0.05, ** *p* < 0.005 and *** *p* < 0.001).

Therefore, to find out these surface characteristics of the QPSF, P_A_, P_N_, P1_AN_, P2_AN_ and P4_AN_ coatings, the equilibrium contact angles between their surface and three pure liquids, i.e., distilled water, ethylene glycol, and diiodomethane (Figure 8 and Figure S16), were measured. Using these values, their surface free energy ($\gamma_{sv}$), as well as the dispersive ($\gamma_{sv}^{d}$) and polar ($\gamma_{sv}^{p}$) components of the free energy were calculated (Table 1) by applying Young [51], Owens and Wendt [52], and Fowkes equations [53] (Equations (3)–(5)).

(3) $$\gamma_{sv}=\gamma_{sl}+\gamma_{lv}\cos\theta$$

(4) $$\gamma_{lv}(1+\cos\theta )=2\sqrt{\gamma_{lv}^{p}\gamma_{sv}^{p}}+2\sqrt{\gamma_{lv}^{d}\gamma_{sv}^{d}}$$

(5) $$\gamma_{sv}=\gamma_{sv}^{d}+\gamma_{sv}^{p}$$

where θ represents the contact angle, $\gamma_{sv}$, $\gamma_{lv}$, and $\gamma_{sl}$ are the surface free energy of the solid (in equilibrium with the liquid’s saturated vapor), the surface free energy of the liquid (in equilibrium with its saturated vapor), and the interfacial free energy between the solid and liquid, respectively; $\gamma_{sv}^{p}$, $\gamma_{lv}^{p}$ and $\gamma_{sv}^{d}$, $\gamma_{lv}^{d}$ are the polar and dispersive components of the surface free energy.

As Figure 8 shows, the surface of the coatings containing one or both drugs had a slightly lower water contact angle value compared to the neat QPSF, indicating a higher degree of wettability. This may be the result of the presence of hydrophilic groups of the drugs, i.e., carboxyl, hydroxyl, or the increase in surface roughness, as indicated by SEM investigation [54]. With the exception of the P2_AN_ sample, all other samples present water contact angle values in the range of moderate wettability, i.e., 60–90°, associated with good biocompatibility, promoting a balance between attraction and repulsion with biological tissues [50]. Furthermore, a moderate wettability promotes a balanced interaction with liquids, which is advantageous in applications like drug delivery or coatings due to the interaction with water without excessive adsorption or resistance, improving the drug release and its bioavailability [55,56].

The surface energy values for the studied coatings and the QPSF reference are summarized in Table 1. The total surface free energy of the QPSF film was measured to be 39.15 mJ/m^2^, with a dominant contribution from the dispersive component and a minimal contribution from the polar component. This is consistent with the low content of quaternary polar sites relative to the longer, less polar polymer chains, as well as the steric hindrance caused by the aliphatic chains. With the exception of the P1_AN_ sample, the drug-loaded coatings exhibited a lower surface energy than QPSF. This decrease is in line with the fine dispersion of the drug molecules within the matrix, in which intermolecular interactions constrain the polymer chains arrangement. Nevertheless, it could be observed that all samples containing amphotericin B (AmB) showed a significant increase in the polar component and a corresponding decrease in the dispersive component of surface energy. This suggests an enhanced presence of polar functional groups on the surface, attributable to the multiple polar hydroxyl groups of the AmB. It can be hypothesised that the drug molecules, particularly AmB, are anchored within the polymeric matrix via London dispersion and van der Waals forces, allowing polar groups to orient themselves toward the surface. In contrast, sample P_N_, which contains norfloxacin (NFX), showed a surface energy dominated by the dispersive component, reflecting the lower content of polar groups relative to AmB (Figure 1). Overall, the surface energy values for all samples fall within the moderate range of 30–300 mJ/m^2^, which is generally considered optimal for promoting moderate cell adhesion and growth [57].

### 3.4. Release Kinetics and Fitting on Mathematical Models

**Figure 9:**
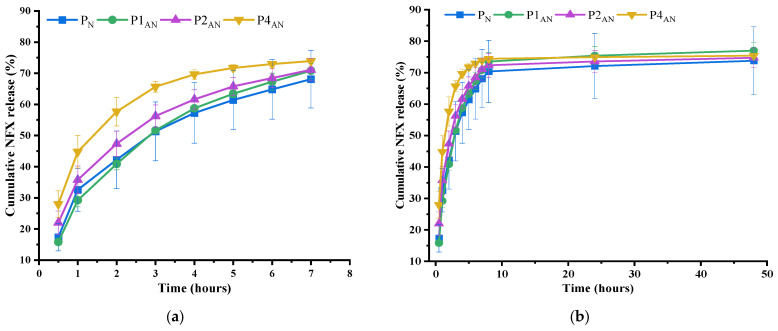
The release kinetics of NFX from P_N_, P1_AN_, P2_AN_, and P4_AN_ coatings (**a**) in the first 8 h and (**b**) over 48 h.

The release kinetics of the drugs was monitored in vitro in PBS (pH 7.4). Despite the high differences in the drug content, the samples demonstrated a similar ability to release NFX, from 75% to 77%, over 48 h, with a faster release in the first 4 h, followed by a slower release (Figure 9a,b). However, the rate of drug release seemed to be controlled by the drug amount,the release rate increasing with the drug content in the matrix. Thus, the P4_AN_ sample released 69% of the encapsulated drug in the first 4 h, while samples P_N_ and P1_AN_ released only ~51%. The different release profile in the first hours can also be correlated with the wettability of the films as it was observed that a higher wettability favored a faster release, consistent with an improved diffusion. It is noteworthy that the presence of AmB in the material structure does not influence the NFX release kinetics, similar values of the percentage of drug released being recorded for both samples P_N_ and P1_AN_. Nevertheless, monitoring the kinetics of AmB release from the polymer matrix was not possible as the supernatant concentration fell below the detection limit of the UV-Vis spectrophotometer (Figure S18). Considering the higher absorbance of AmB compared to NFX for similar concentrations, it can be appreciated that AmB was released in very low amounts, most probably due to its low solubility (0.02 mg/mL).

Generally, the release kinetic of a drug from a polymeric matrix is governed by one or more mechanisms that are influenced by the chemical structure of the matrix, its hydrophilic or hydrophobic nature, and morphology [58], as well as the interactions between the matrix and the drug [59]. Therefore, in order to identify the parameters/factors which dictate the NFX release from the P_N_, P_A_, P1_AN_, P2_AN_, and P4_AN_ coatings, the values obtained from the in vitro release experiments were fitted on five mathematical models for two distinct stages: 1–5 h and 6–48 h, respectively (Figure S19). Following the plotting of the experimental release data on the zero-order model, high values of the correlation coefficient were obtained (R^2^ = 0.95–0.97%) in the first stage for all the samples except P4_AN_ (R^2^ = 0.9). This data (Table 2) revealed that the release of NFX is influenced only by time, and the process occurs at a constant rate independent of the amount of drug from matrix. The value of the K_0_ constant, which reflects the release rate, is lower for P4_AN_ than for other samples, a fact that can be correlated to a steeper concentration gradient between the drug matrix and the surrounding environment, which might initially drive a faster release and that could then slow down as the drug amount decreases. The higher NFX concentration in P4_AN_, which corresponds to a greater number of functional groups, resulted in enhanced interactions between the drug and the matrix, effectively anchoring the drug within the polymeric matrix and slowing its diffusion [59]. However, according to the results of water contact angle determination, this sample presented the most hydrophilic character, which may contribute to the diffusion of the drug in the first hours of exposure to the buffer solution. Further, the experimental drug release data were plotted in accordance with the first-order equation, and the graphical representation led to a line with a correlation coefficient between 0.94 and 0.99. These results highlight the fact that, in the first stage, the release of the NFX is influenced also by the amount of drug dispersed within the polymeric matrix. The best results in terms of correlation coefficient were obtained for all the samples during the first stage by fitting the experimental data on Higuchi and Korsmeyer–Peppas models, with R^2^ values close to unity (R^2^ = 0.95–0.99 for Higuchi and R^2^ = 0.97–0.99 for Korsmeyer–Peppas). By applying the Higuchi model, it was noticed that the release process of NFX from the quaternized polysulfone matrix is dictated by the diffusion of the drug molecules through the matrix. The Korsmeyer–Peppas equation revealed the type of drug diffusion based on the values of release exponent, n. All the samples exhibited a Fickian diffusion, according to which the rate of solvent transport or diffusion, greatly exceeds the rate of polymeric chain relaxation. Even if the Fickian diffusion generally takes place in polymeric matrices with a vitreous transition temperature (T_g_) lower than room temperature, this phenomenon also occurs in the case of polymers with T_g_ higher than the ambient temperature when a plasticizing agent is added to the system [60]. In this study, no plasticizing agent was added to the casting solution of the coatings, but we can corelate the occurrence of this type of diffusion to the spacer role of the dimethyl butyl ammonium group, anchored to the main chain of polysulfone structure, as well as to the polar nature of materials that facilitate a good interaction of the buffer solution with the matrix. The experimental data were also represented according to the Hixson–Crowell cube root law, and the correlation coefficient obtained was greater than 0.93 in the first stage, which suggests that erosion and diffusion processes occurred simultaneously. To summarize, the release of NFX from the polymeric matrix is a diffusion-controlled process governed by Fickian transport, which is influenced by the amount of drug, time of exposure, and hydrophilicity of the matrix.

### 3.5. Antioxidant Activity Using the DPPH Method

**Figure 10:**
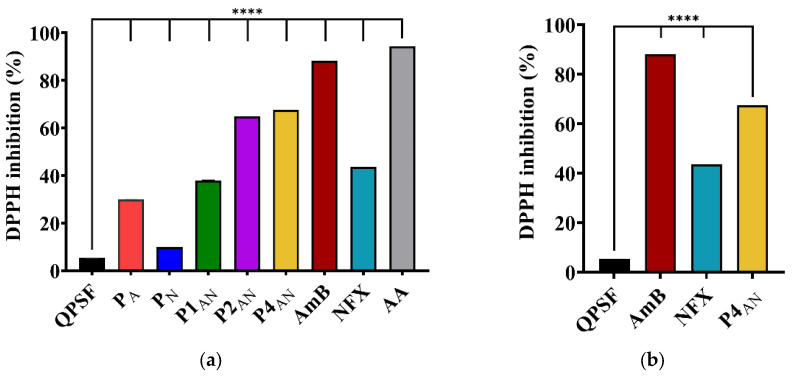
(**a**) Antioxidant activity of the samples investigated in this study, along with three controls (ascorbic acid, amphotericin B and norfloxacin) and (**b**) comparative antioxidant activity of P4_AN_ and its components—QPSF, AmB, and NFX (**** *p* < 0.0001).

AmB exhibits seven conjugated double bonds in its structure, which probably makes the drug itself susceptible to autoxidation, leading to an antioxidant effect. This behavior is similar to that of carotenoids and retinoids [61]. This drug is a chain-breaking antioxidant; its mechanism of action consists in shortening the length of the oxidation chain following its reaction with radicals [62]. Moreover, the chemical structure of AmB shows that it can interact with DPPH radicals, either through its hydroxyl groups, which may serve as hydrogen donors to neutralize free radicals, or through its polyene chain, which is capable of stabilizing unpaired electrons and thereby facilitating radical scavenging. On the other hand, NFX has been shown to enhance the antioxidant activity and diminish the infection-induced oxidative stress, in line with the presence of functional groups able to inhibit free radicals, such as carboxyl, carbonyl, and secondary amine [63]. Furthermore, quaternary ammonium group may scavenge reactive oxidative species (ROS) and reduce lipid oxidation [64]. Starting from these premises, ROS scavenging effect of the prepared coatings was investigated by the DPPH method, and the results are presented in Figure 10. As expected, the QPSF films showed low antioxidant activity, with a radical scavenging ability of 5.3%, consistent with the scarce exposure to the surface of the quaternary ammonium groups. In line with the literature data, free AmB and NFX displayed high antioxidant activity (88.19% and 43.64%) due to the presence of the functional groups capable of binding radical species and the good mobility of the molecules in solution. Their good activity was also preserved in the coatings at lower levels, probably guided by their slow diffusion through the polymeric matrix, leading to low drug concentration in the experiment conditions. The presence of AmB induced an obvious increase of the sample’s ability to inhibit the free radicals along its content in the coatings, with a threshold at 67.49%, possibly corelated with solubility limit of the drug (0.1 mg/mL in methanol). The coatings containing both drugs displayed higher activity compared to those containing a single drug at similar concentration, indicating the cumulation of their effects. The radical scavenging activity of coatings was significantly improved by the addition of AmB and NFX to the polymeric matrix. Therefore, the RSA increased from 5.3% to 29.94% and from 5.3% to 10.02% by adding AmB or NFX to QPSF, respectively. The highest antioxidant activity was observed in the case of the P4_AN_ sample, 67.49%, which can be correlated to the additive antioxidant effect of the two drugs dispersed within the polymeric matrix.

### 3.6. Antimicrobial Activity

Considering that the aim of this study was to develop antimicrobial coatings through an intimate dispersion of two drugs with complementary activity, a broad-spectrum antibiotic and an antifungal drug, in a quaternized polysulfone matrix, we subsequently investigated their antimicrobial activity against relevant bacteria and fungi frequently associated with hospital-acquired infections, namely *S. aureus* (Gram-positive bacteria), *E. coli* (Gram-negative bacteria) and *C. albicans* (opportunistic microorganisms). We used two different in vitro antimicrobial tests to observe (*i*) the viability of microorganisms on the surface of the materials or (*ii*) the capacity of the formulations to inhibit the growth of bacteria and fungi following their exposure in contact with the investigated materials.

**Figure 11:**
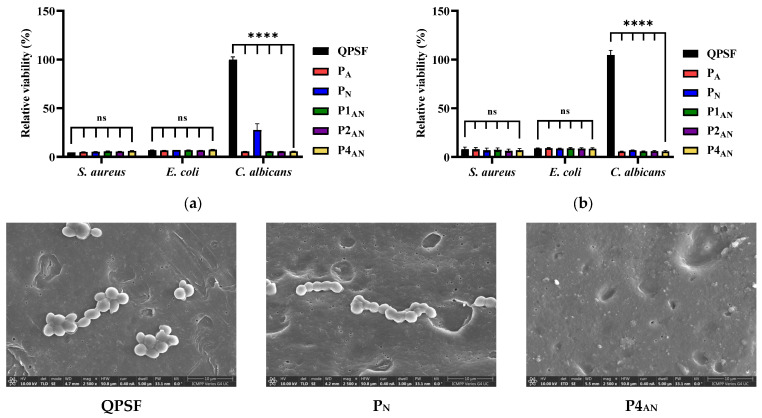
Bacteriostatic and fungistatic activity of the formulations against the three reference strains evaluated by (**a**) MTS and (**b**) OD600 assays and representative SEM images obtained after the interaction with *Candida albicans* (scale: 10 µm). **** *p* < 0.0001.

As Figure 11 shows, almost all samples showed bacteriostatic and fungistatic activity against the tested reference strains. No significant differences were observed between the two methods (MTS and OD600 assays) used to quantify the microbial adherence. All the samples showed high efficiency against the Gram-positive and Gram-negative bacterial strain represented by *S. aureus* (between 5 and 8% of cell viability) and *E. coli* (between 4 and 10%), regardless of the sample composition, indicating the major contribution of the quaternary ammonium groups in the polymer matrix, justified by its significant higher content compared to drugs (Table S2). In the case of *C. albicans*, it can be observed that the samples without AmB in their composition, namely QPSF and P_N_, favored the adhesion of this fungal strain on their surface, registering a cell viability of 99.86% for QPSF and 27.67% for P_N_, and the difference between the QPSF and all the other samples was statistically significant (Figure 11). This observation was further supported by SEM images, which revealed adhered yeast cells on the surface of QPSF, while the surface of P4AN appeared clean and free of microbial attachment.

**Figure 12:**
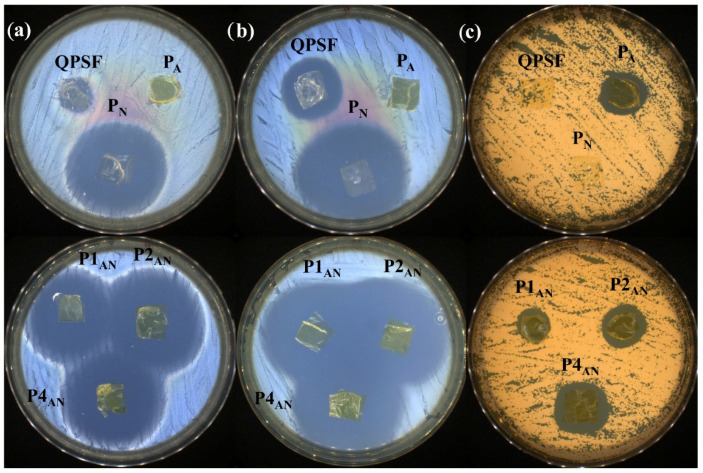
Antimicrobial activity of the investigated samples against: (**a**) *S. aureus*, (**b**) *E. coli* and (**c**) *C. albicans*.

Although microbial adhesion tests provide information regarding the direct interaction between microorganisms and the material surface, as well as on the ability of coatings to prevent microbial colonization, which plays an important role in preventing infections associated with medical devices, a comprehensive evaluation of the antimicrobial activity also requires the evaluation of the release efficiency of encapsulated antimicrobial agents. To this end, the diffusimetric method was employed, and the obtained data are presented in Table 3 and Figure 12. The analysis of these data offers a clear understanding of each component’s contribution to the overall antimicrobial effect.

The plain QPSF exhibited inherent antibacterial activity, showing moderate efficacy against *Staphylococcus aureus* (inhibition diameter up to 14 mm) and stronger activity against *Escherichia coli* (up to 23 mm). However, no antifungal activity was observed against *Candida albicans*.

Encapsulation of AmB in sample P_A_ induced antifungal activity (inhibition diameter up to 18 mm), but interestingly, no antibacterial activity was detected. This loss can be attributed to the reduced mobility of QPSF chains, resulting from stronger intermolecular interactions with AmB. Such interactions could limit QPSF diffusion in the microbiological medium, thereby impairing its antibacterial performance.

Conversely, encapsulation of NFX in the P_N_ sample significantly enhanced the antibacterial activity compared to the plain QPSF sample, consistent with the observed NFX release. As expected, no antifungal activity was recorded in the absence of an antifungal agent.

When both antimicrobial agents, NFX and AmB, were co-encapsulated, the resulting coatings exhibited dual antimicrobial activity, showing inhibition diameters that were comparable to those of the P_A_ and P_N_ samples. This confirms that both drugs were released in sufficient concentrations to inhibit bacterial and fungal growth. Moreover, the progressive drug release likely provides a sustained barrier against microbial colonization, regardless of the drug content in the coating, as supported by NFX release kinetics.

Although in vitro release of AmB could not be quantified as its concentration remained below the UV-Vis detection limit, the observed antifungal activity in the AmB-containing sample (P_A_) strongly suggests that the drug was released from the polymer matrix at levels sufficient to exert a therapeutic effect. The pronounced inhibition zones recorded for all drug-loaded samples highlights their ability to provide a functional antimicrobial barrier against pathogenic microorganisms.

## 4. Conclusions

The present study evaluated the possibility of using quaternized polysulfone as a matrix for encapsulating antimicrobial agents in order to obtain coatings for medical devices or surgical instruments that are capable of preventing infections which may occur at the surgical site. Five different coatings were prepared by dispersing amphotericin B (AmB) and/or norfloxacin (NFX) into the quaternized polysulfone, to reach 1% *w/w*, 2% *w/w*, and 4% *w/w*. ^1^H-NMR, FTIR, and UV-Vis spectroscopy showed strong physical interactions between the components, which favoured a fine dispersion of drugs into the matrix, confirmed by the absence of specific reflections in XRD diffractograms. In addition, POM images suggested an interface-coupled ordering, attributed to the electrostatic interactions between the positively charged amino groups of the polymer and the electron-rich groups of the drugs. The investigated materials presented smooth surface and a compact internal structure, with rare nanometric aggregation. The water contact angle presented values in the range of 70–90°, which corresponds to a moderate wetting range. By adding the two drugs to the material structure, the radical oxidant scavenging capacity increases from 5.3% (QPSF) to 67.49% (P4_AN_), a property that can be attributed to the additive effect of the antioxidant activity of the two drugs. The norfloxacin release profile showed a progressive release, with higher speed in the first four hours, especially for the sample which contains the highest amount of drug. By fitting the release data on five different mathematical models, it was established that the release process is a diffusion-controlled one, influenced by the amount of drug and the hydrophilicity of the material. The release of Amphotericin B could not be effectively monitored using the UV-Vis method, likely due to its slow-release rate, low encapsulated amount, and limited solubility. Nevertheless, the samples containing AmB exhibited enhanced antifungal activity against *C. albicans*, supporting its efficacy even at extremely low concentrations. Furthermore, the tested materials demonstrated strong antimicrobial activity against all three reference strains, *S. aureus*, *E. coli*, and *C. albicans*, highlighting their potential as antimicrobial coatings for surgical devices to prevent microbial colonization.

## Figures and Tables

**Table 1:** Surface free energy values and components for the investigated coatings in mJ/m^2^.

Sample	$\gamma_{sv}^{d}$	$\gamma_{sv}^{p}$	$\gamma_{sv}$
QPSF	22.36	16.79	39.15
P_A_	0.47	37.49	37.96
P_N_	13.96	7.06	21.02
P1_AN_	1.50	43.39	44.89
P2_AN_	4.95	11.63	16.58
P4_AN_	0.96	36.10	37.06

**Table 2:** NFX release kinetics parameters for the first (1–5 h) and second stage (6–48 h), resulting from fitting the in vitro NFX release and applying different mathematical models.

Code	Zero Order		First Order		Higuchi		Hixson–Crowell		Korsmeyer–Peppas		
	R^2^	K_0_	R^2^	K_1_	R^2^	K_H_	R^2^	k	R^2^	k	n
	1st stage										
P_N_	0.97	7.27	0.99	−0.14	0.99	23.89	0.99	−0.18	0.99	32.47	0.4
P1_AN_	0.97	8.62	0.99	−0.16	0.99	28.35	0.99	−0.21	0.99	29.33	0.5
P2_AN_	0.95	7.42	0.98	−0.15	0.99	24.53	0.98	−0.19	0.99	36.12	0.38
P4_AN_	0.90	6.58	0.94	−0.16	0.95	22.04	0.93	−0.19	0.97	45.84	0.3
	2nd stage										
P_N_	0.66	0.47	0.99	0.009	–	–	0.69	−0.006	–	–	–
P1_AN_	0.64	0.5	0.99	0.012	–	–	0.67	−0.006	–	–	–
P2_AN_	0.65	0.49	0.91	0.02	–	–	0.67	−0.004	–	–	–
P4_AN_	0.67	0.29	0.75	0.051	–	–	0.68	−0.002	–	–	–

R^2^—correlation coefficient; K—proportionality constant; n_r_—release exponent.

**Table 3:** Antimicrobial activity (mm) of the tested samples against the reference strains.

Sample	Inhibition Zone (mm)		
	*S. aureus*	*E. coli*	*C. albicans*
QPSF	14.33 ± 0.46	22.56 ± 0.32	-
P_A_	-	-	17.63 ± 0.15
P_N_	32.63 ± 0.66	36.53 ± 0.55	-
P1_AN_	31.96 ± 0.30	37.90 ± 0.62	13.86 ± 0.05
P2_AN_	32.73 ± 1.00	39.67 ± 0.45	16.63 ± 0.51
P4_AN_	33.40 ± 0.26	39.40 ± 0.17	20.86 ± 0.83

## Data Availability

The original contributions presented in this study are included in the article/Supplementary Materials. Further inquiries can be directed to the corresponding author(s).

## Supplementary Materials

The following supporting information can be downloaded at: https://www.mdpi.com/article/10.3390/polym17131869/s1, References [65,66,67,68] are cited in the Supplementary Materials. Figure S1. ^1^H-NMR spectrum of CMPSF. Figure S2. ^1^H-NMR spectrum of QPSF. Figure S3. ^13^C-NMR spectrum of QPSF. Figure S4. H,H-COSY NMR spectrum of QPSF. Figure S5. H,C-HSQC NMR spectrum of QPSF. Figure S6. H,C-HMBC spectrum of QPSF. Figure S7. ^1^H-NMR spectra of 5 distinct samples from each coating recorded in order to determine the statistical significance of the chemical shift of the protons in the methylene bridge as a function of composition. Figure S8. Graphical representation of the chemical shift characteristic to the methylene bridge proton (around 4.61 (left) −4.52 (right)). Figure S9. ^1^H-NMR spectra of P4_AN_ sample at different temperatures (21–50 °C). Figure S10. FTIR spectra of PSF, CMPSF and QPSF. Figure S11. Deconvolution of the bands between 3700–3100 cm^−1^ spectral region. Figure S12. UV spectra of amphotericin B at different molar concentration in PBS. Figure S13. Representative SEM images of QPSF and P4_AN_ samples. Figure S14. EDAX mapping of the samples showing the homogeneous distribution of the elements and consequently a homogeneous distribution of the drugs into the coatings. Figure S15. SEM images at a larger scale 200 µm (inset cross section). Figure S16. Images of contact angle of water, ethylene glycol (EG) and diiodomethane (DIM) on the surface of the studied samples. Figure S17. Calibration curve of NFX. Figure S18. UV spectra of P2_AN_ sample recorded at different periods of time for the drug release kinetics. Figure S19. Linear forms of the Higuchi (a), Korsmeyer-Peppas (b), zero-order (c and d), first-order (e and f) and Hixon (g and h) models applied for the release of NFX from the investigates materials for the two release stages. Table S1. EDAX analysis of the samples. Table S2. The molar ratio of the components into the investigated samples.

## References

[1] Jain S., Nehra M., Kumar R., Dilbaghi N., Hu T.Y., Kumar S., Kaushik A., Li C.-Z. (2021). Internet of Medical Things (IoMT)-Integrated Biosensors for Point-of-Care Testing of Infectious Diseases. Biosens. Bioelectron..

[2] Jiang X., Yao Y., Tang W., Han D., Zhang L., Zhao K., Wang S., Meng Y. (2020). Design of Dental Implants at Materials Level: An Overview. J. Biomed. Mater. Res. A.

[3] Qu Y., McGiffin D., Hayward C., McLean J., Duncan C., Robson D., Kure C., Shen R., Williams H., Mayo S. (2020). Characterization of Infected, Explanted Ventricular Assist Device Drivelines: The Role of Biofilms and Microgaps in the Driveline Tunnel. J. Heart Lung Transplant..

[4] Salwiczek M., Qu Y., Gardiner J., Strugnell R.A., Lithgow T., McLean K.M., Thissen H. (2014). Emerging Rules for Effective Antimicrobial Coatings. Trends Biotechnol..

[5] Devine R., Douglass M., Ashcraft M., Tayag N., Handa H. (2021). Development of Novel Amphotericin B-Immobilized Nitric Oxide-Releasing Platform for the Prevention of Broad-Spectrum Infections and Thrombosis. ACS Appl. Mater. Interfaces.

[6] de Lissovoy G., Fraeman K., Hutchins V., Murphy D., Song D., Vaughn B.B. (2009). Surgical Site Infection: Incidence and Impact on Hospital Utilization and Treatment Costs. Am. J. Infect. Control.

[7] Harder E.E., Gaies M.G., Yu S., Donohue J.E., Hanauer D.A., Goldberg C.S., Hirsch J.C. (2013). Risk Factors for Surgical Site Infection in Pediatric Cardiac Surgery Patients Undergoing Delayed Sternal Closure. J. Thorac. Cardiovasc. Surg..

[8] Saito Y., Kobayashi H., Uetera Y., Yasuhara H., Kajiura T., Okubo T. (2014). Microbial Contamination of Surgical Instruments Used for Laparotomy. Am. J. Infect. Control.

[9] Cooper I.R., Pollini M., Paladini F. (2016). The Potential of Photo-Deposited Silver Coatings on Foley Catheters to Prevent Urinary Tract Infections. Mater. Sci. Eng. C.

[10] Lim K., Chua R.R.Y., Ho B., Tambyah P.A., Hadinoto K., Leong S.S.J. (2015). Development of a Catheter Functionalized by a Polydopamine Peptide Coating with Antimicrobial and Antibiofilm Properties. Acta Biomater..

[11] Singha P., Locklin J., Handa H. (2017). A Review of the Recent Advances in Antimicrobial Coatings for Urinary Catheters. Acta Biomater..

[12] Czerwińska-Główka D., Przystaś W., Zabłocka-Godlewska E., Student S., Cwalina B., Łapkowski M., Krukiewicz K. (2021). Electrically-Responsive Antimicrobial Coatings Based on a Tetracycline-Loaded Poly(3,4-Ethylenedioxythiophene) Matrix. Mater. Sci. Eng. C.

[13] Pinho A.C., Piedade A.P. (2020). Polymeric Coatings with Antimicrobial Activity: A Short Review. Polymers.

[14] Filimon A., Olaru N., Doroftei F., Coroaba A., Dunca S. (2021). Processing of Quaternized Polysulfones Solutions as Tool in Design of Electrospun Nanofibers: Microstructural Characteristics and Antimicrobial Activity. J. Mol. Liq..

[15] Bargan A., Onofrei M.D., Stoica I., Doroftei F., Dunca S., Filimon A. (2022). Materials Based on Quaternized Polysulfones with Potential Applications in Biomedical Field: Structure–Properties Relationship. Int. J. Mol. Sci..

[16] Dumbrava O., Filimon A., Marin L. (2023). Tailoring Properties and Applications of Polysulfone Membranes by Chemical Modification: Structure-Properties-Applications Relationship. Eur. Polym. J..

[17] Chen Y., Lin B., Qiu Y. (2023). Modification of Polysulfone and the Biomedical Application of Modified Polysulfone. Int. J. Polym. Mater. Polym. Biomater..

[18] Wasyłeczko M., Wojciechowski C., Chwojnowski A. (2024). Polyethersulfone Polymer for Biomedical Applications and Biotechnology. Int. J. Mol. Sci..

[19] Sivaraman K.M., Kellenberger C., Pané S., Ergeneman O., Lühmann T., Luechinger N.A., Hall H., Stark W.J., Nelson B.J. (2012). Porous Polysulfone Coatings for Enhanced Drug Delivery. Biomed. Microdevices.

[20] Giles C., Lamont-Friedrich S.J., Michl T.D., Griesser H.J., Coad B.R. (2018). The Importance of Fungal Pathogens and Antifungal Coatings in Medical Device Infections. Biotechnol. Adv..

[21] Cyphert E.L., von Recum H.A. (2017). Emerging Technologies for Long-Term Antimicrobial Device Coatings: Advantages and Limitations. Exp. Biol. Med..

[22] Salmanton-García J., Cornely O.A., Stemler J., Barać A., Steinmann J., Siváková A., Akalin E.H., Arikan-Akdagli S., Loughlin L., Toscano C. (2024). Attributable Mortality of Candidemia—Results from the ECMM Candida III Multinational European Observational Cohort Study. J. Infect..

[23] Wisplinghoff H., Seifert H., Wenzel R.P., Edmond M.B. (2006). Inflammatory Response and Clinical Course of Adult Patients with Nosocomial Bloodstream Infections Caused by *Candida* spp.. Clin. Microbiol. Infect..

[24] Marsh P.K., Tally F.P., Kellum J., Callow A., Gorbach S.L. (1983). Candida Infections in Surgical Patients. Ann. Surg..

[25] Laniado-Laborín R., Cabrales-Vargas M.N. (2009). Amphotericin B: Side Effects and Toxicity. Rev. Iberoam. Micol..

[26] Vitale R.G., Afeltra J., de Hoog G.S., Rijs A.J., Verweij P.E. (2003). In Vitro Activity of Amphotericin B and Itraconazole in Combination with Flucytosine, Sulfadiazine and Quinolones against Exophiala Spinifera. J. Antimicrob. Chemother..

[27] Ghimici L., Avram E. (2003). Viscometric Behavior of Quaternized Polysulfones. J. Appl. Polym. Sci..

[28] Ren Y., Huang L., Wang Y., Mei L., Fan R., He M., Wang C., Tong A., Chen H., Guo G. (2020). Stereocomplexed Electrospun Nanofibers Containing Poly (Lactic Acid) Modified Quaternized Chitosan for Wound Healing. Carbohydr. Polym..

[29] Ailincai D., Cibotaru S., Anisiei A., Coman C.G., Pasca A.S., Rosca I., Sandu A.I., Mititelu-Tartau L., Marin L. (2023). Mesoporous Chitosan Nanofibers Loaded with Norfloxacin and Coated with Phenylboronic Acid Perform as Bioabsorbable Active Dressings to Accelerate the Healing of Burn Wounds. Carbohydr. Polym..

[30] (2010). Antibacterial Products—Test for Antibacterial Activity and Efficacy.

[31] Bauer A.W., Perry D.M., Kirby W.M.M. (1959). Single-Disk Antibiotic-Sensitivity Testing of Staphylococci; an Analysis of Technique and Results. AMA Arch. Intern. Med..

[32] Sandström K., Wärmländer S., Leijon M., Gräslund A. (2003). 1H NMR Studies of Selective Interactions of Norfloxacin with Double-Stranded DNA. Biochem. Biophys. Res. Commun..

[33] Rajini Balakrishnan A., Easwaran K.R.K. (1993). CD and NMR Studies on the Aggregation of Amphotericin-B in Solution. Biochim. Biophys. Acta (BBA)—Biomembr..

[34] Alkhouzaam A., Qiblawey H. (2021). Novel Polysulfone Ultrafiltration Membranes Incorporating Polydopamine Functionalized Graphene Oxide with Enhanced Flux and Fouling Resistance. J. Membr. Sci..

[35] Sahoo S., Chakraborti C.K., Behera P.K., Mishra S.C. (2012). FTIR and Raman Spectroscopic Investigations of a Norfloxacin/Carbopol934 Polymeric Suspension. J. Young Pharm..

[36] Gagoś M., Arczewska M. (2011). Influence of K^+^ and Na^+^ Ions on the Aggregation Processes of Antibiotic Amphotericin B: Electronic Absorption and FTIR Spectroscopic Studies. J. Phys. Chem. B.

[37] Vandermeulen G., Rouxhet L., Arien A., Brewster M.E., Préat V. (2006). Encapsulation of Amphotericin B in Poly(Ethylene Glycol)-Block-Poly(ε-Caprolactone-Co-Trimethylenecarbonate) Polymeric Micelles. Int. J. Pharm..

[38] Chang Y., Wang Y.H., Hu C.Q. (2011). Simultaneous Determination of Purity and Potency of Amphotericin B by HPLC. J. Antibiot..

[39] Das I., Halder M. (2017). Counterpointing Scenarios on the Fate of Different Prototropic Forms of Norfloxacin Housed in the Pocket of Lysozyme: The Nonelectrostatic Interactions in the Protein Interior Are in the Controlling Role on the Prototropic Equilibria of the Guest. ACS Omega.

[40] Ailincai D., Gavril G., Marin L. (2020). Polyvinyl Alcohol Boric Acid—A Promising Tool for the Development of Sustained Release Drug Delivery Systems. Mater. Sci. Eng. C.

[41] Vinodh R., Purushothaman M., Sangeetha D. (2011). Novel Quaternized Polysulfone/ZrO2 Composite Membranes for Solid Alkaline Fuel Cell Applications. Int. J. Hydrogen Energy.

[42] Soto R., Patel P., Albadarin A.B., Diniz M.O., Hudson S.P. (2022). Solubility, Aggregation and Stability of Amphotericin B Drug in Pure Organic Solvents: Thermodynamic Analysis and Solid Form Characterization. J. Mol. Liq..

[43] Bratu I., Borodi G., Kacsó I., Moldovan Z., Filip C., Dragan F., Vasilescu M., Simon S. (2011). New Solid Form of Norfloxacin: Structural Studies. J. Spectrosc..

[44] Lekoane T., Msomi P.F. (2023). Quarternized Polysulfone/ZSM-5 Zeolite Composite Anion Exchange Membrane Separators for Aluminum-Air Battery. J. Appl. Polym. Sci..

[45] Marin L., Ailincai D., Paslaru E. (2014). Monodisperse PDLC Composites Generated by Use of Polyvinyl Alcohol Boric Acid as Matrix. RSC Adv..

[46] Ailincai D., Dorobanu A.M., Dima B., Irimiciuc T.A., Lupacu C., Agop M., Olguta O. (2020). Poly(Vinyl Alcohol Boric Acid)-Diclofenac Sodium Salt Drug Delivery Systems: Experimental and Theoretical Studies. J. Immunol. Res..

[47] Menzies K.L., Jones L. (2010). The Impact of Contact Angle on the Biocompatibility of Biomaterials. Optom. Vis. Sci..

[48] Kumar S., Bhushan P., Bhattacharya S. (2017). Coatings on Surgical Tools and How to Promote Adhesion of Bio-Friendly Coatings on Their Surfaces. Adhesion in Pharmaceutical, Biomedical and Dental Fields.

[49] Alias R., Mahmoodian R., Abd Shukor M.H. (2019). Development and Characterization of a Multilayer Silver/Silver-Tantalum Oxide Thin Film Coating on Stainless Steel for Biomedical Applications. Int. J. Adhes. Adhes..

[50] Ikada Y. (1994). Surface Modification of Polymers for Medical Applications. Biomaterials.

[51] Young T. (1805). III. An Essay on the Cohesion of Fluids. Philos. Trans. R. Soc. Lond..

[52] Owens D.K., Wendt R.C. (1969). Estimation of the Surface Free Energy of Polymers. J. Appl. Polym. Sci..

[53] Fowkes F.M. (1964). Contact Angle, Wettability, and Adhesion. Adv. Chem..

[54] Pathote D., Kumari P., Singh V., Jaiswal D., Gautam R.K., Behera C.K. (2023). Biocompatibility Evaluation, Wettability, and Scratch Behavior of Ta-Coated 316L Stainless Steel by DC Magnetron Sputtering for the Orthopedic Applications. Surf. Coat. Technol..

[55] La Zara D., Zhang F., Sun F., Bailey M.R., Quayle M.J., Petersson G., Folestad S., van Ommen J.R. (2021). Drug Powders with Tunable Wettability by Atomic and Molecular Layer Deposition: From Highly Hydrophilic to Superhydrophobic. Appl. Mater. Today.

[56] Singh D., Dilip Saoji S. (2024). The Role of Surface Energy and Wettability in Polymer-Based Drug Delivery Systems: Enhancing Bioadhesion and Drug Release Efficiency. J. Macromol. Sci. Part B Phys..

[57] Majhy B., Priyadarshini P., Sen A.K. (2021). Effect of Surface Energy and Roughness on Cell Adhesion and Growth—Facile Surface Modification for Enhanced Cell Culture. RSC Adv..

[58] Paarakh M.P., Jose P.A., Setty C., Peterchristoper G.V. (2018). Release Kinetics—Concepts and Applications. Int. J. Pharm. Res. Technol. (IJPRT).

[59] Ailincai D., Morariu S., Rosca I., Sandu A.I., Marin L. (2023). Drug Delivery Based on a Supramolecular Chemistry Approach by Using Chitosan Hydrogels. Int. J. Biol. Macromol..

[60] (2015). Mathematical Models of Drug Release. Strategies to Modify the Drug Release from Pharmaceutical Systems.

[61] Osaka K., Tyurina Y.Y., Dubey R.K., Tyurin V.A., Ritov V.B., Quinn P.J., Branch R.A., Kagan V.E. (1997). Amphotericin B as an Intracellular Antioxidant: Protection against 2,2′-Azobis(2,4-Dimethylvaleronitrile)-Induced Peroxidation of Membrane Phospholipids in Rat Aortic Smooth Muscle Cells. Biochem. Pharmacol..

[62] Kancheva V.D., Dettori M.A., Fabbri D., Alov P., Angelova S.E., Slavova-Kazakova A.K., Carta P., Menshov V.A., Yablonskaya O.I., Trofimov A.V. (2021). Natural Chain-Breaking Antioxidants and Their Synthetic Analogs as Modulators of Oxidative Stress. Antioxidants.

[63] Alam R.T.M., Fawzi E.M., Alkhalf M.I., Alansari W.S., Aleya L., Abdel-Daim M.M. (2018). Anti-Inflammatory, Immunomodulatory, and Antioxidant Activities of Allicin, Norfloxacin, or Their Combination against Pasteurella Multocida Infection in Male New Zealand Rabbits. Oxid. Med. Cell. Longev..

[64] Cui J., Sun Y., Wang L., Ji Y., Zhao H., Sun M., Guo Z., Dong F. (2025). Quaternary Ammonium Salts of Chitosan Containing Aromatic Ring: Synthesis, Characterization, Antimicrobial, Antioxidant and Cytotoxicity. Carbohydr. Res..

[65] Yang J., Li Q., Oluf J., Pan C., Cleemann L.N., Bjerrum N.J., He R. (2012). Phosphoric Acid Doped Imidazolium Polysulfone Membranes for High Temperature Proton Exchange Membrane Fuel Cells. J Power Sources.

[66] Yang J., Wang J., Liu C., Gao L., Xu Y., Che Q., He R. (2015). Influences of the Structure of Imidazolium Pendants on the Properties of Polysulfone-Based High Temperature Proton Conducting Membranes. J. Membr. Sci..

[67] Liu Y., Wang J. (2020). Preparation of Anion Exchange Membrane by Efficient Functionalization of Polysulfone for Electrodialysis. J. Membr. Sci..

[68] Avram E. (2001). Polymers with Pendent Functional Groups. VI. A Comparative Study on the Chloromethylation of Linear Polystyrene and Polysulfone with Paraformaldehyde/Me3SiCl. Polym. Plast. Technol. Eng..

