# Supplementary material for: Quaternized Polysulfones as Matrix for the Development of Broad-Spectrum Antimicrobial Coatings for Medical Devices
Source: Polymers (Basel). 2025 Jul 3;17(13):1869. doi: 10.3390/polym17131869 (PMC12251730; doi:10.3390/polym17131869)
Supplement: Supplementary file 1 [file polymers-17-01869-s001.zip › polymers-3721950-supplementary.pdf]

Supporting material for

Quaternized Polysulfones as Matrix for the Development of Broad-Spectrum Antimicrobial Coatings for Medical Devices

Oana Dumbrava, Irina Rosca, Daniela Ailincai * and Luminita Marin *

"Petru Poni" Institute of Macromolecular Chemistry, 700487 Iasi, Romania; dumbrava.oana@icmpp.ro (O.D.); rosca.irina@icmpp.ro (I.R.)
* Correspondence: ailincai.daniela@icmpp.ro (D.A.); lmarin@icmpp.ro (L.M.)

Synthesis of CMPSF

Synthesis of QPSF

Structural characterization of CMPSF and QPSF

Figure S1. ^{1}H-NMR spectra of CMPSF

Figure S2. ^{1}H-NMR spectrum of QPSF

Figure S3. ^{13}C-NMR spectrum of QPSF

Figure S4. H,H-COSY NMR spectrum of QPSF

Figure S5. H,C-HSQC NMR spectrum of QPSF

Figure S6. H,C-HMBC spectrum of QPSF

Figure S7. ^{1}H-NMR spectra of 5 distinct samples from each coating recorded in order to determine the statistical significance of the chemical shift of the protons in the methylene bridge as a function of composition

Figure S8. Graphical representation of the chemical shift characteristic to the methylene bridge proton (around 4.61 (left) - 4.52 (right))

Figure S9. ^{1}H-NMR spectra of $P4_{AN}$ sample at different temperatures (21-50 °C)

Figure S10. FTIR spectra of PSF, CMPSF and QPSF

Figure S11. Deconvolution of the bands between 3700-3100 cm^{-1} spectral region

Figure S12. UV spectra of amphotericin B at different molar concentration in PBS

Figure S13. Representative SEM images of QPSF and $P4_{AN}$ samples

Figure S14. EDAX mapping of the samples showing the homogeneous distribution of the elements and consequently a homogeneous distribution of the drugs into the coatings

Figure S15. SEM images at a larger scale 200 µm (inset cross section)

Figure S16. Images of contact angle of water, ethylene glycol (EG) and diiodomethane (DIM) on the surface of the studied samples

Figure S17. Calibration curve of NFX

Figure S18. UV spectra of $P2_{AN}$ sample recorded at different periods of time for the drug release kinetics

Figure S19. Linear forms of the Higuchi (a), Korsmeyer-Peppas (b), zero-order (c and d), first- order (e and f) and Hixon (g and h) models applied for the release of NFX from the investigates materials for the two release stages.

Table S1. EDAX analysis of the samples

Table S2. The molar ratio of the components into the investigated samples

Synthesis of CMPSF

Chloromethylated polysulfone (CMPSF) was used as a precursor in order to obtain quaternized polysulfone (QPSF). To this aim, a first step in the synthetic pathway of QPSF is represented by the chloromethylation of polysulfone structure. Therefore, CMPSF was synthesized following a protocol described in the literature [1–4], with several modifications. PSF was dissolved in chloroform and stirred for half of hour to obtain a homogeneous solution. Trimethyl chlorosilane, paraformaldehyde and anhydrous tin tetrachloride were added to the polysulfone solution in a molar ratio of 1:15:15:0.23. The reaction took proceeded for 96 hours, at 50 °C, in nitrogen atmosphere to avoid the occurrence of side reactions. The CMPSF was isolated from the reaction mixture by precipitation in methanol, on an ice bath, and filtration. Further, the synthesis product was washed with water and methanol and dried under vacuum at 40 °C for 24 hours. ^{1}H-NMR (400.13 MHz, $CDCl_3$, δ, ppm, Figure S1): 7.87-7.84 (m, H-3C, H-3D), 7.36 (s, H-3'B), 7.24 (d, J=8.4 Hz, H-3A), 7.16 (d, J=8.4 Hz, H-3B), 7.03-7.00 (m, H-2C, H-2D), 6.95-6.94 (m, H-2A), 6.84 (d, J=8.4 Hz, H-2B), 4.54 (s, CH_2-Cl), 1.70 (s, (-$C(CH_3)_2$).

Synthesis of QPSF

CMPSF was dissolved in DMF and N,N-dimethylbutyl amine (DMBA) was added dropwise to the CMPSF solution. The reaction mixture was stirred at 60 °C for 48 hours. Once the synthesis was completed, QPSF was purified by concentration of the reaction mixture on a rotary evaporator, followed by precipitation in acetone, filtration and drying under vacuum for 48 hours. ^{1}H-NMR (400.13 MHz, DMSO-d_6, δ, ppm, Figure S1): 8.01-7.92 (m, H-3C, H-3D), 7.77 (s, H-3'B, monosubstituted), 7.63 (s. H-3'B, disubstituted), 7.46-7.43 (m, H-3B), 7.34-7.13 (m, H-3A, H-2C, H-2D), 7.06-7.03 (m, H-2A, H-2B, H-2'B), 4.64 (s, H5, disubstituted), 4.56 (s, H5 disubstituted), 3.28 (br s, H6), 3.03-2.99 (H10), 1.70 (H7, $C(CH_3)_2$), 1.18-1.13 (m, H8), 0.86-0.81 (m, H9).

Structural characterization of CMPSF and QPSF

^{1}H-NMR and FTIR spectra confirmed the successful synthesis of chloromethylated and quaternized polysulfone, by the appearance of the specific signals for each functionalized polymer. By comparing the ^{1}H-NMR spectrum of the chloromethylated polysulfone (Figure S1) and pristine polysulfone, it can be observed the appearance of new signals in the ^{1}H-NMR spectrum of CMPSF, namely: a sharp signal at 4.54 ppm attributed to the two protons from the –CH_2Cl group and three new signals at 7.36, 7.17 and 6.84 ppm which correspond to the protons from the substituted aromatic ring B. The substitution degree (SD) of CMPSF was calculated with the aid of equation S1 and it was equal to 1.4 chloromethyl groups per monomeric unit.

$$SD = \frac{I_{CH_2Cl}}{I_{CH_3}} \cdot 3 \tag{S1}$$

where I_{CH_2Cl} is the integral value corresponding to the (–CH_2Cl) peak at 4.54 ppm and I_{CH_3} is the integral value corresponding to the isopropyl protons peak at 1.70 ppm.

In the ^{1}H-NMR spectrum of the quaternized polysulfone (Figure S2) appear new signals at 3.28, 3.03-2.99, 1.18-1.13, 1.67 (overlapped with the signal for -$C(CH_3)_2$ in bisphenol fragment) and 0.86-0.81 ppm, assigned to the protons of newly introduced dimethylbutylammonium group. Moreover, in the region characteristic to the aromatic protons can be observed the peaks assigned to the protons from the substituted aromatic rings which are located at 7.70 (H3' B monosubstituted),7.63 ppm (H3' AB disubstituted), 7.44 ppm (H3B), 7.03-7.12 ppm (H2B and H2'B overlapped with H2A). The SD was determined from ^{1}H-NMR by applying the following equation (2) and was found to be 1.4 dimethylbutulammonium groups per monomeric units, the same as DS for the starting CMPSF:

$$SD = \frac{I_{H_5}}{I_{H_3CD}} \cdot 2 \tag{S2}$$

where I_{H_5} is the integral value for the CH_2N protons (4.56-4.64 ppm) and I_{H_3CD} is the integral value for H3 (8.01-7.92 ppm) from the diphenyl sulfone unit.

The successful synthesis of QPSF was also confirmed by FTIR spectroscopy. Thus, in the FTIR spectra of QPSF (Figure S10), one can observe the appearance of new absorption bands around 3395cm^{-1}, characteristic of hydrogen bonds that can form with atmospheric water due to its hygroscopic nature, as well as absorption bands located between 2850 and 2966 cm^{-}

[1] that correspond to the vibrations of the $-CH_2-$ and CH_3 groups of the dimethylbutylammonium residue grafted onto the polysulfone backbone.

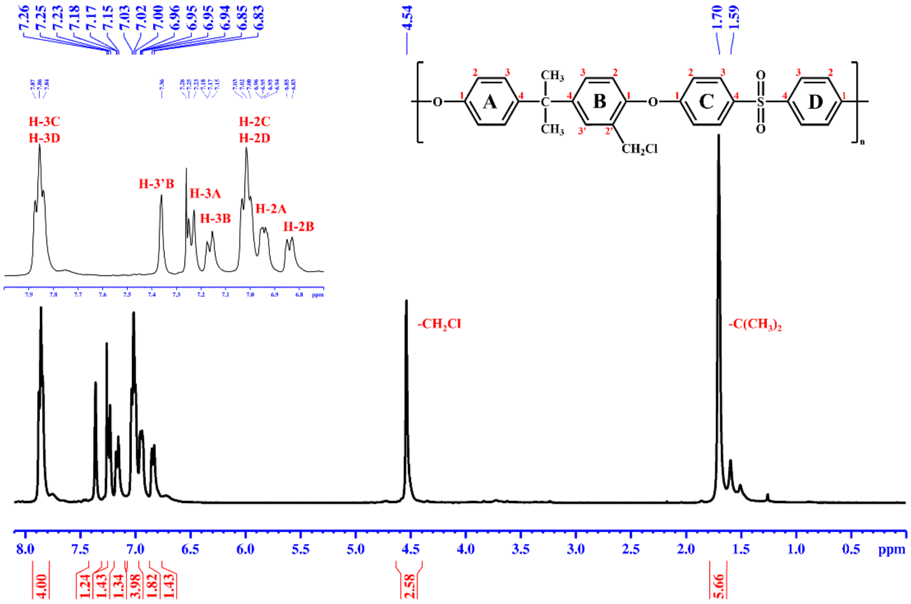

Figure S1. ^{1}H-NMR spectrum of CMPSF

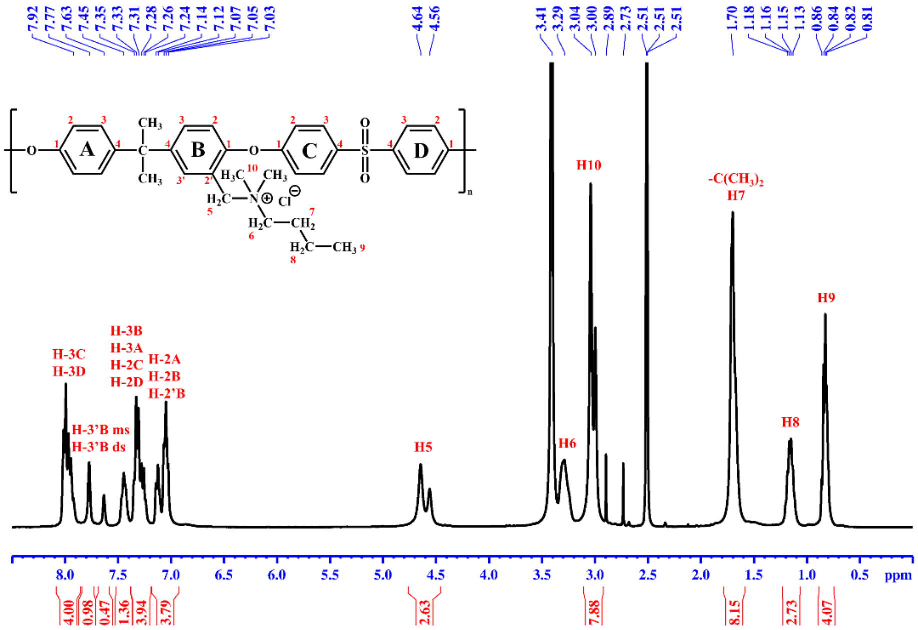

Figure S2. ^{1}H-NMR spectrum of QPSF

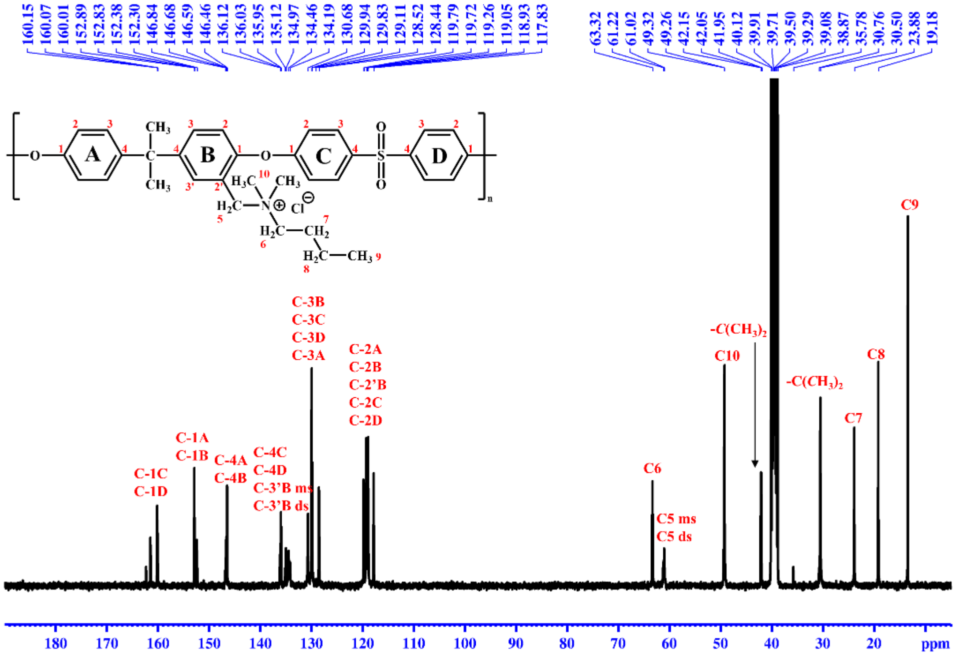

Figure S3. ^{13}C-NMR spectrum of QPSF

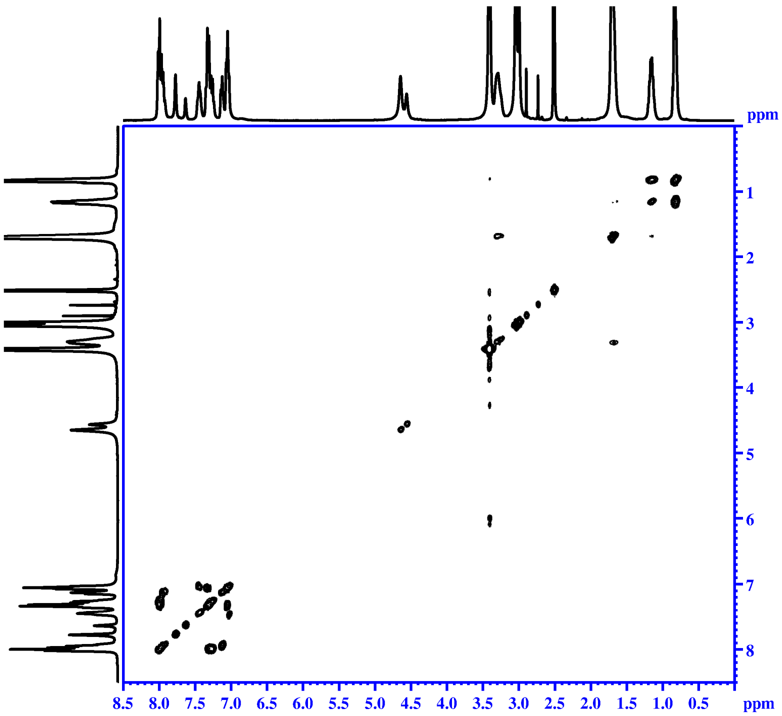

Figure S4. H,H-COSY NMR spectrum of QPSF

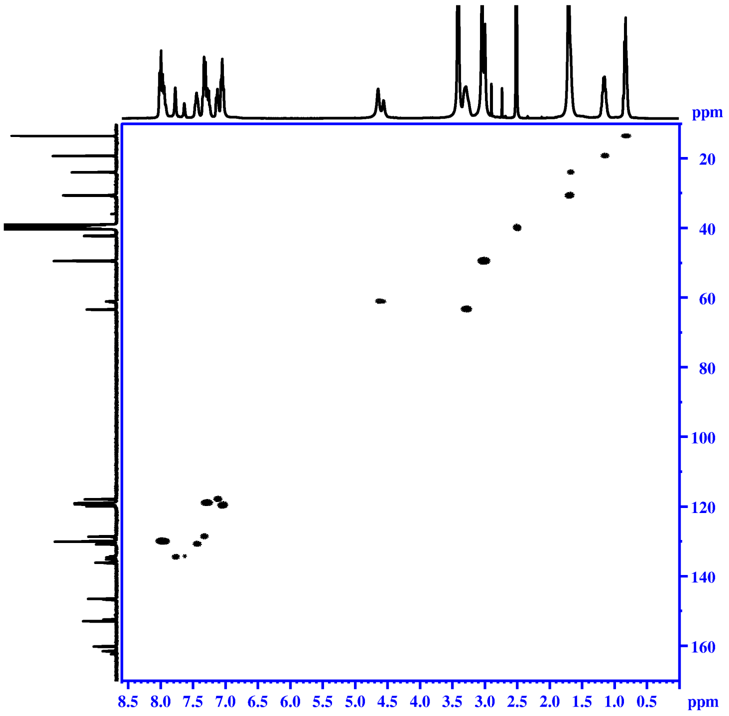

Figure S5. H,C-HSQC NMR spectrum of QPSF

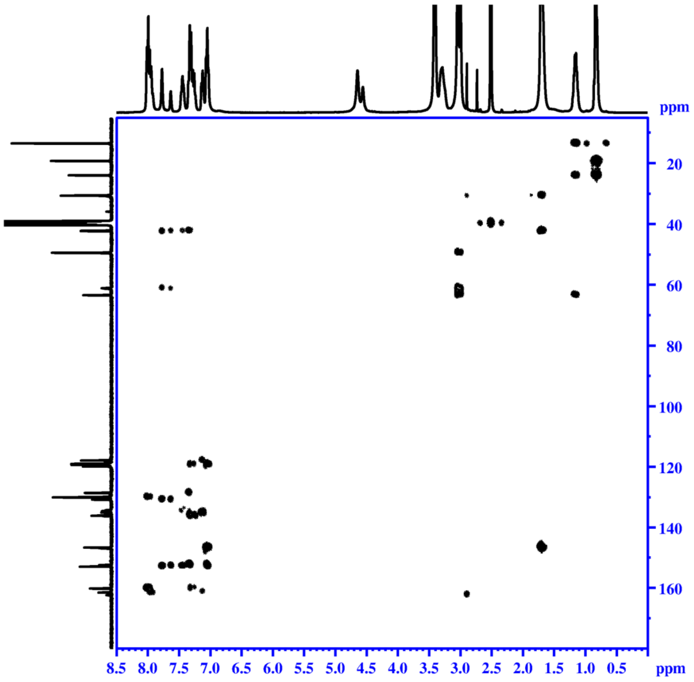

Figure S6. H,C-HMBC spectrum of QPSF

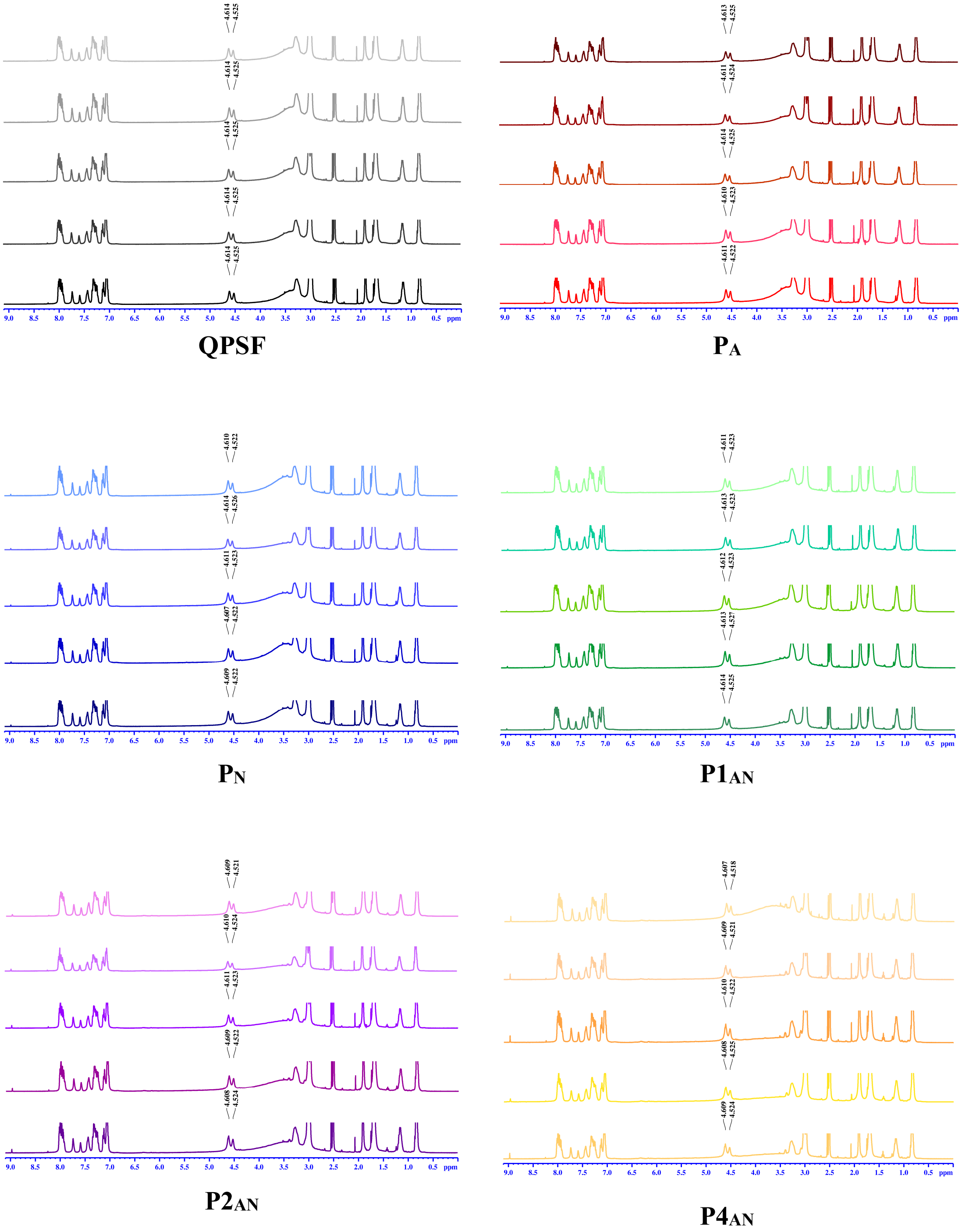

Figure S7. ^{1}H-NMR spectra of 5 distinct samples from each coating recorded in order to determine the statistical significance of the chemical shift of the protons in the methylene bridge as a function of composition

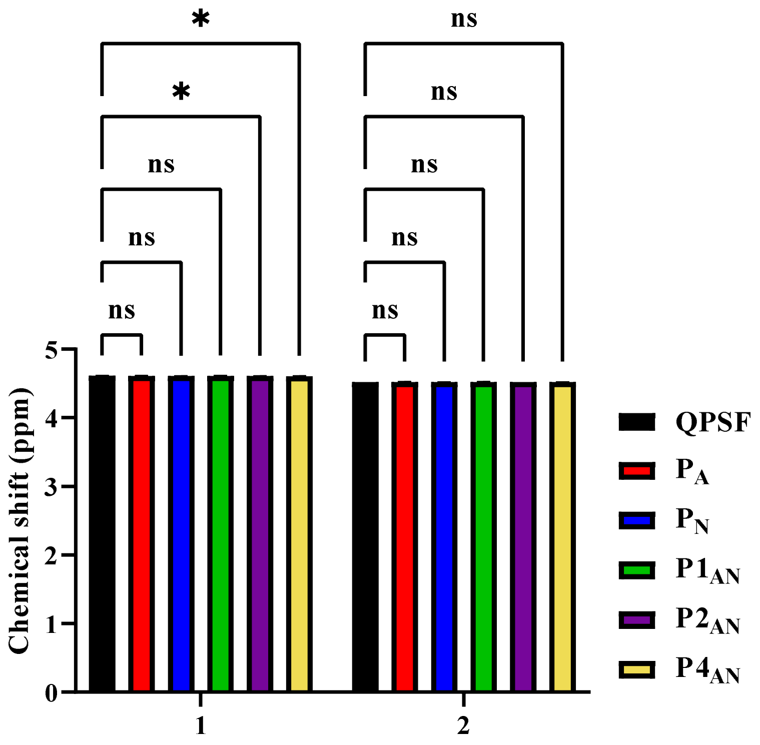

Figure S8. Graphical representation of the chemical shift characteristic to the methylene bridge proton (around 4.61 (left) - 4.52 (right))

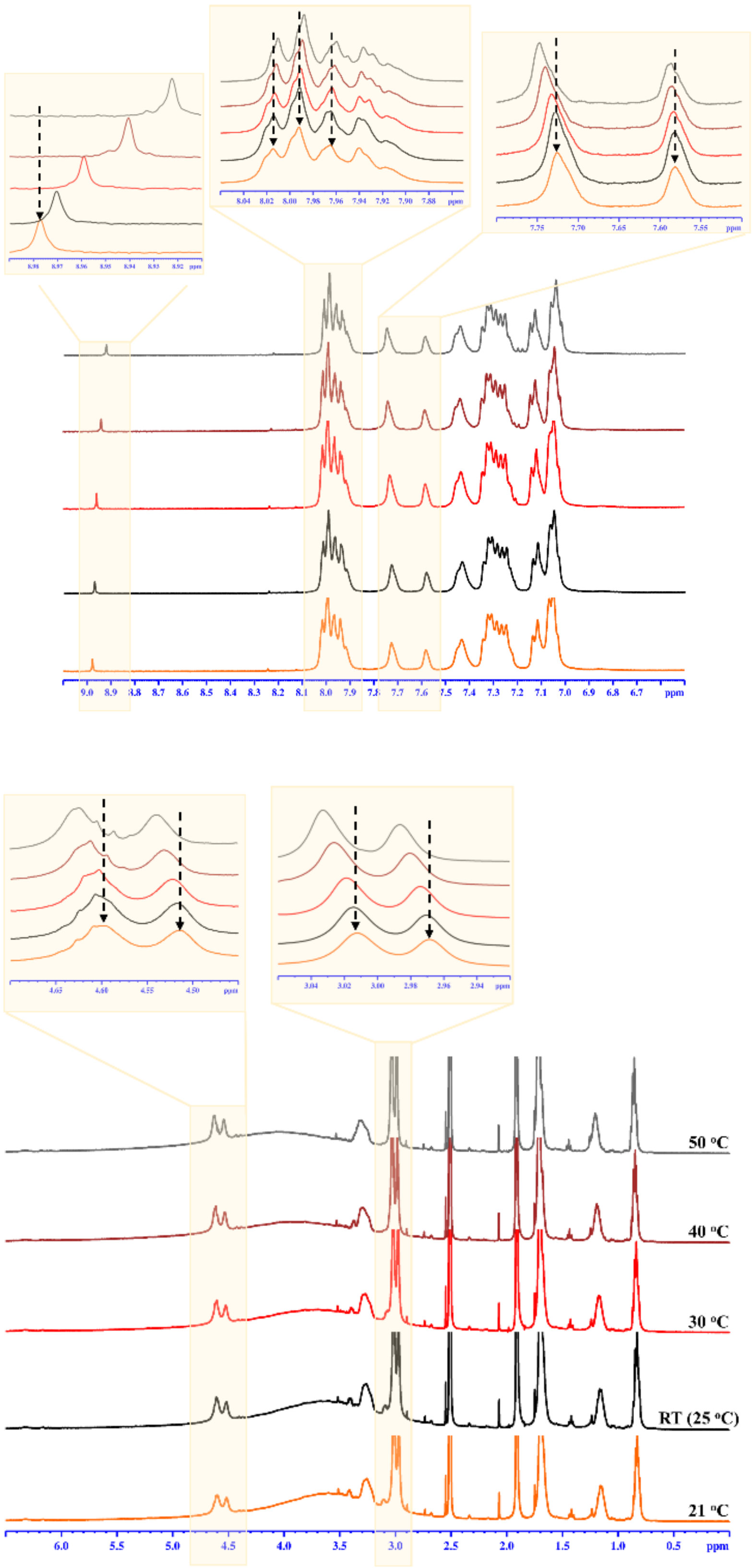

Figure S9. ^{1}H-NMR spectra of $P4_{AN}$ sample at different temperatures (21-50 °C)

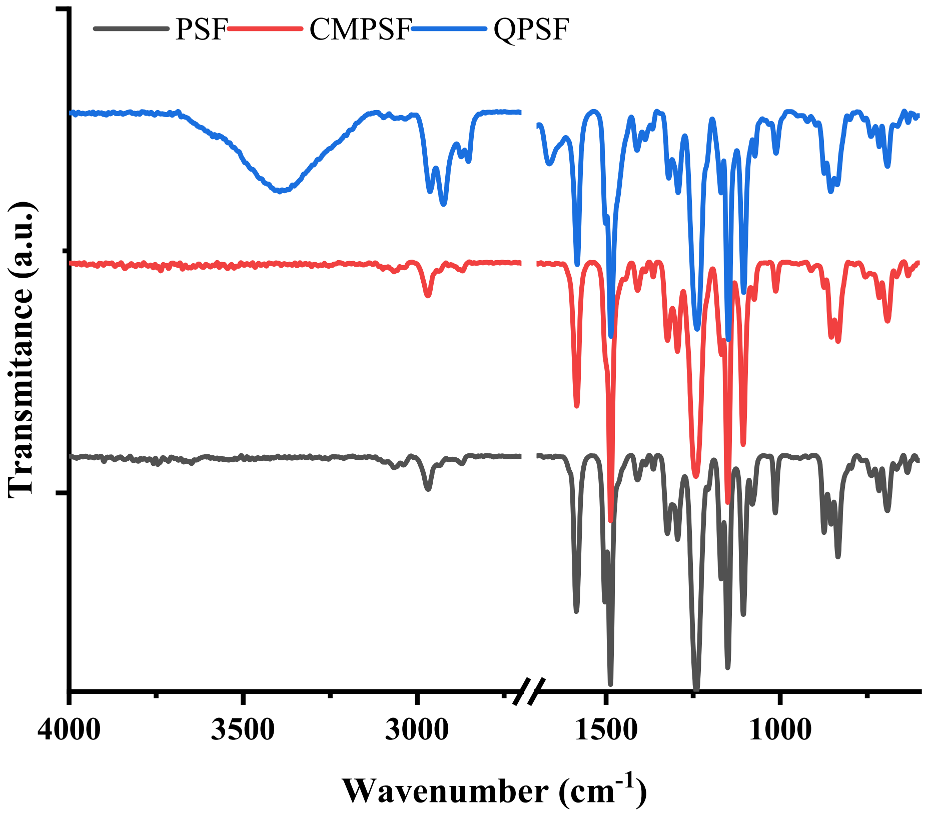

Figure S10. FTIR spectra of PSF, CMPSF and QPSF

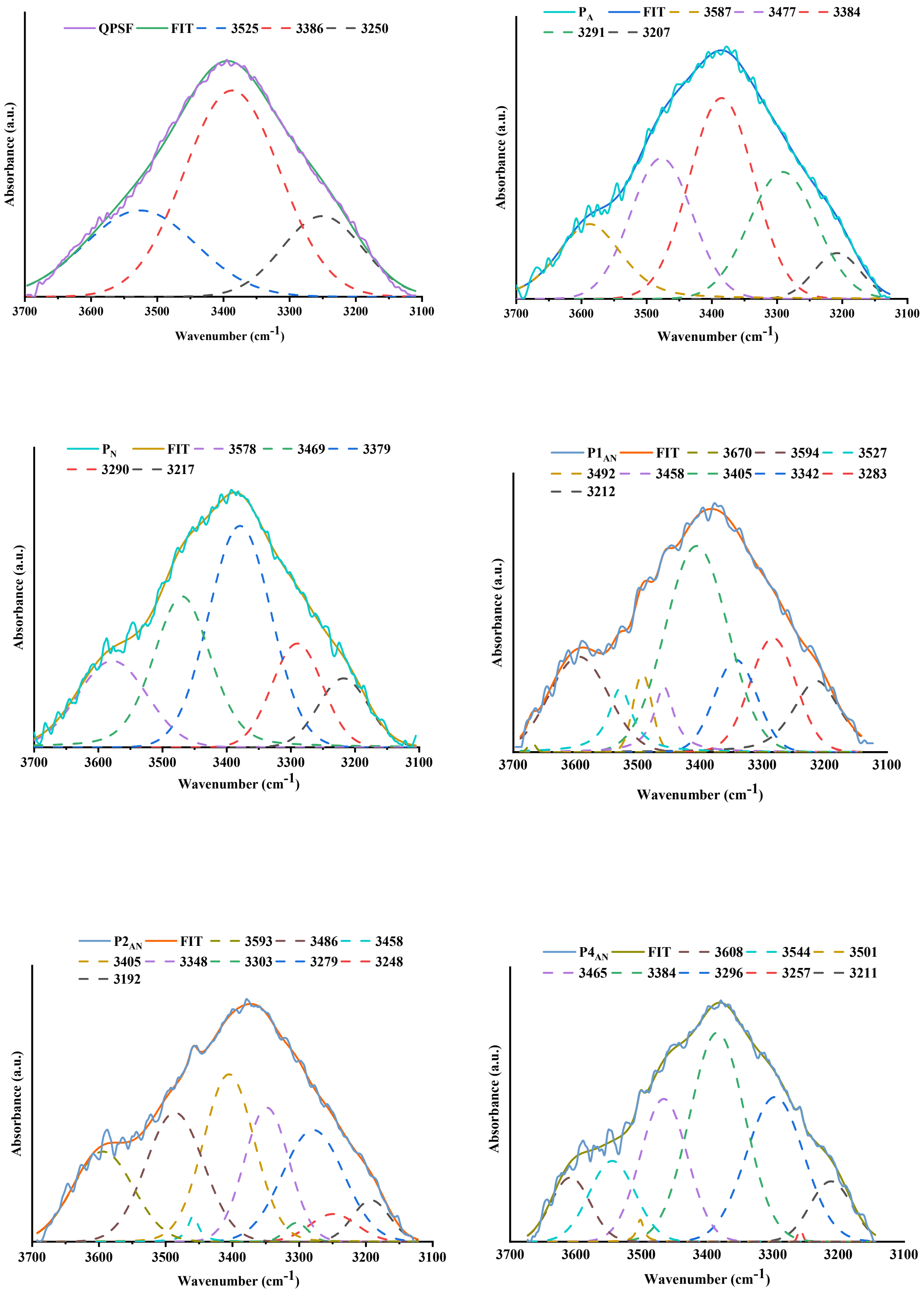

Figure S11. Deconvolution of the bands between 3700-3100 cm^{-1} spectral region

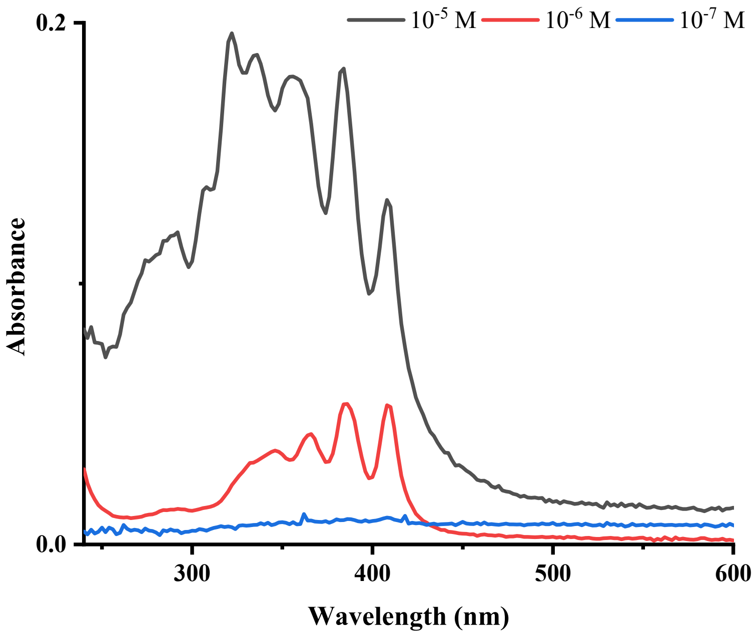

Figure S12. UV spectra of amphotericin B at different molar concentration in PBS

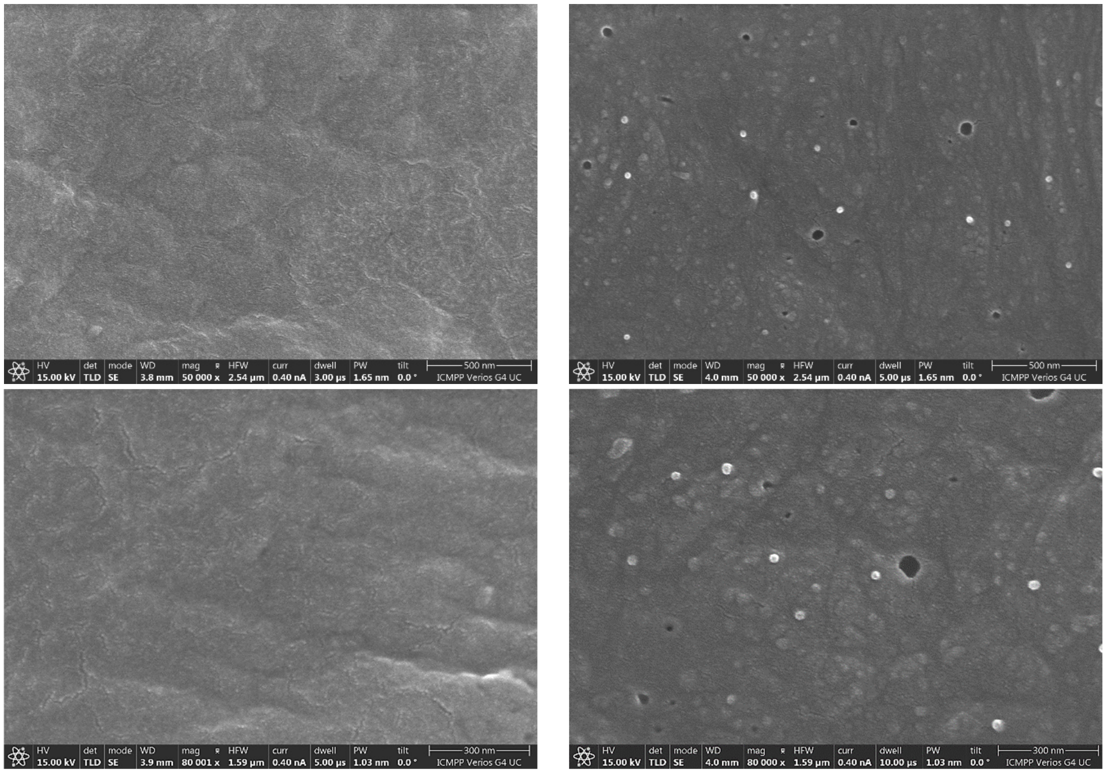

QPSF **P4$_{AN}$**

Figure S13. Representative SEM images of QPSF and P4$_{AN}$ samples

QPSF

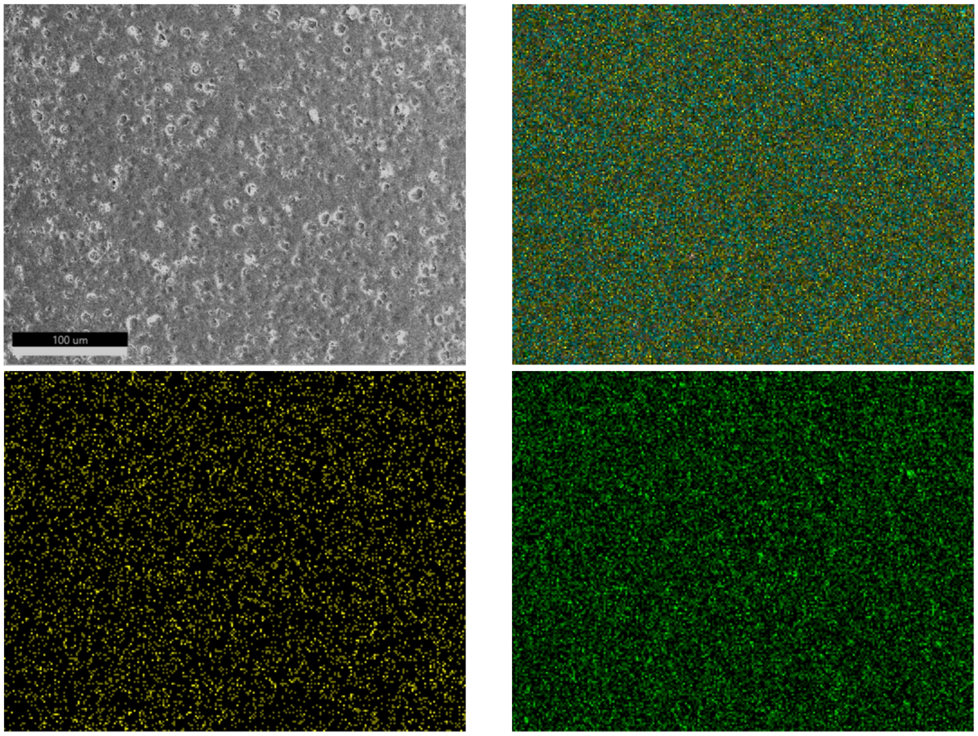

Nitrogen Oxygen

P_A

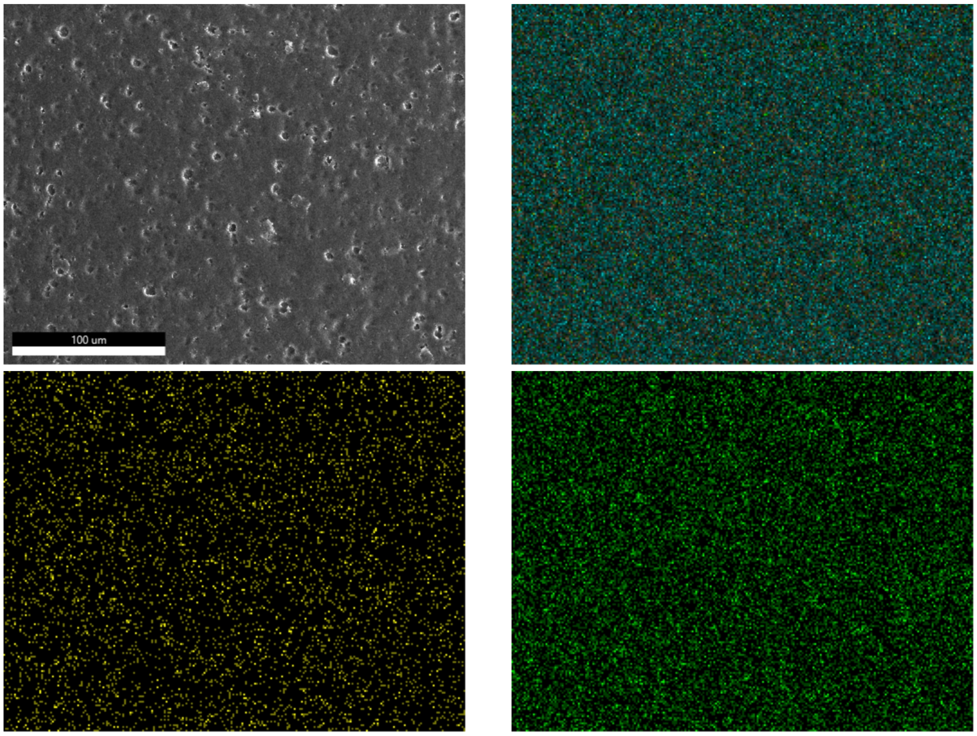

Nitrogen Oxygen

P_N

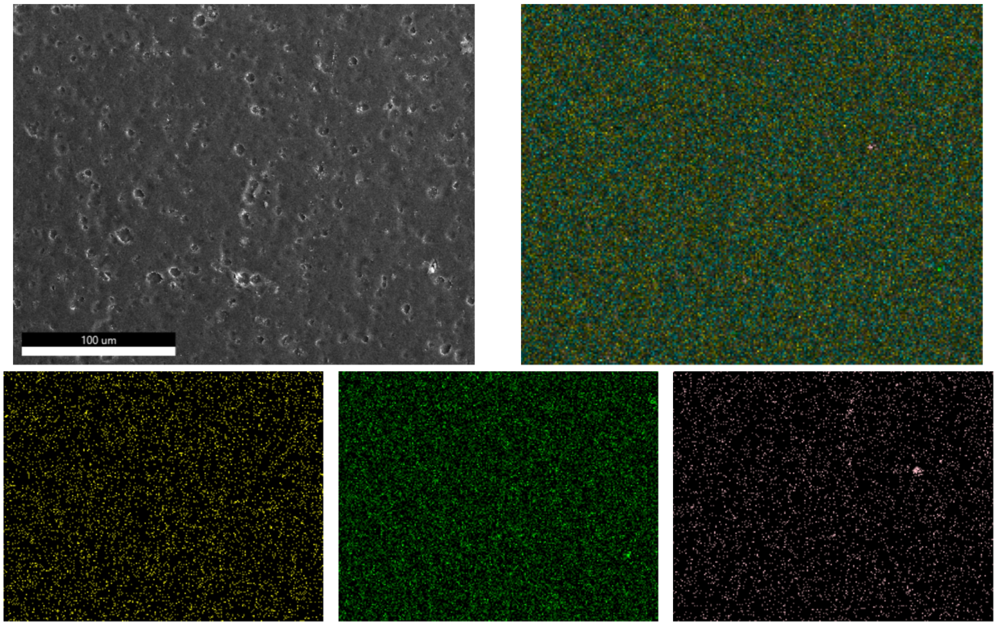

Nitrogen Oxygen Fluorine

$P1_{AN}$

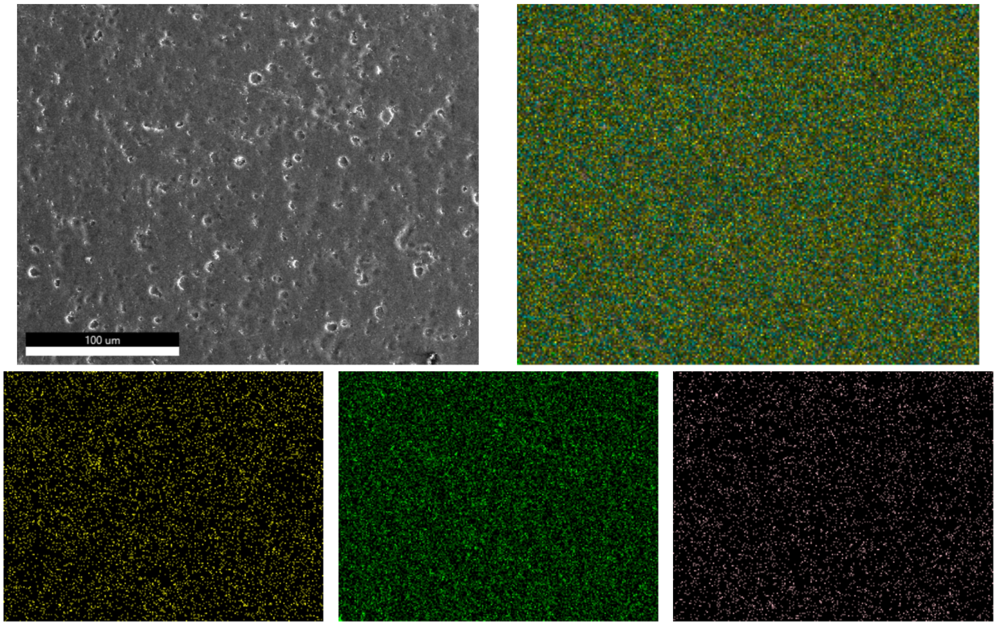

Nitrogen Oxygen Fluorine

$P2_{AN}$

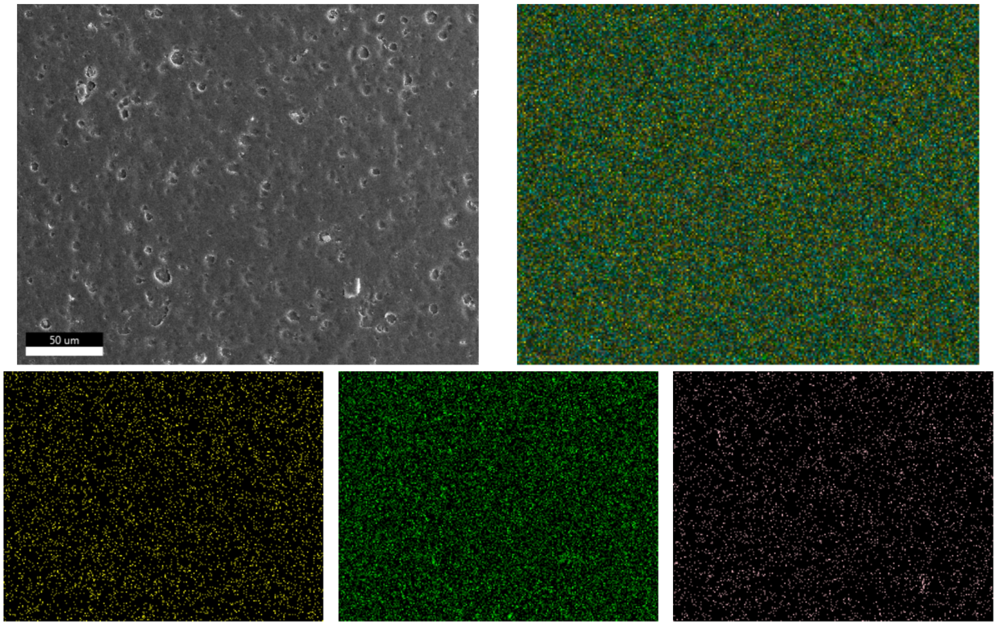

Nitrogen Oxygen Fluorine

$P4_{AN}$

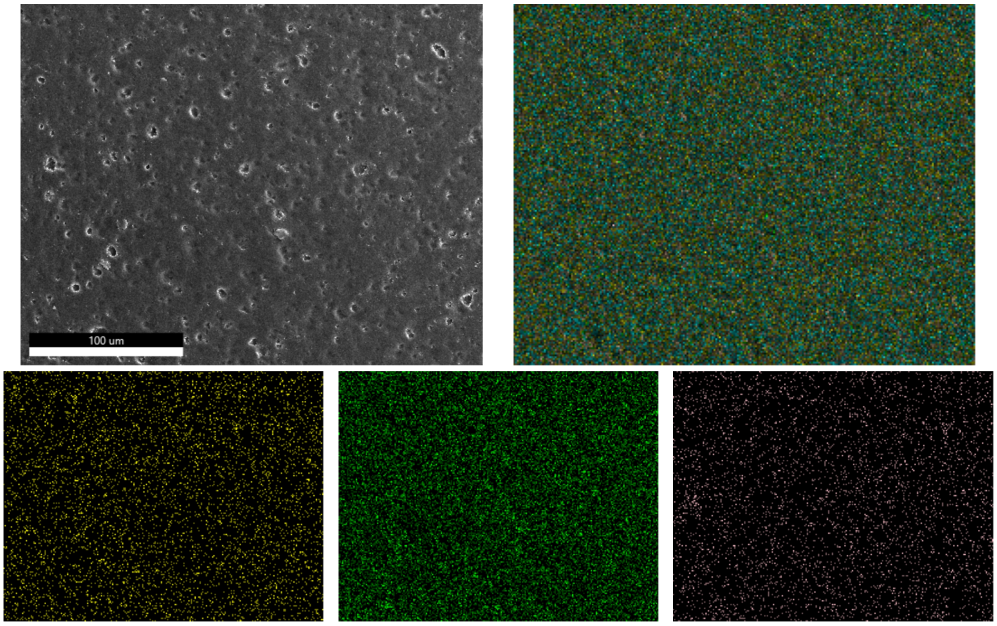

Nitrogen Oxygen Fluorine

Figure S14. EDAX mapping of the samples showing the homogeneous distribution of the elements and consequently a homogeneous distribution of the drugs into the coatings

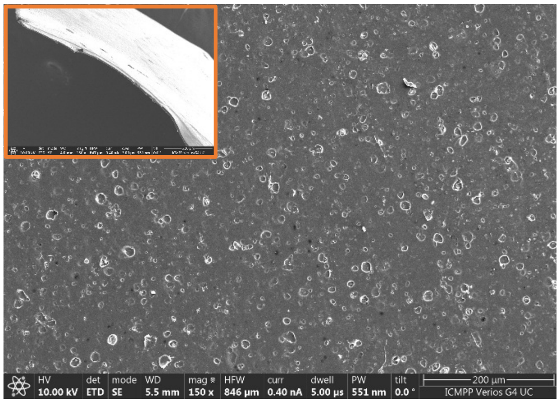

a)

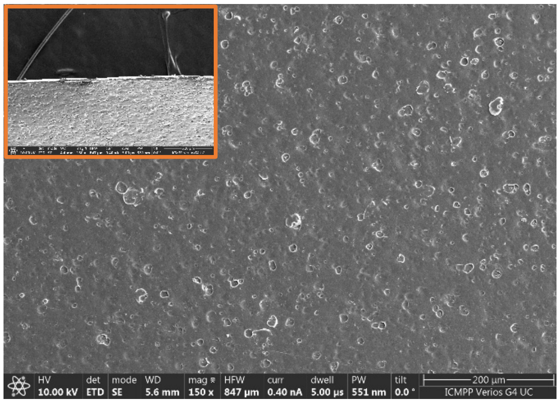

b)

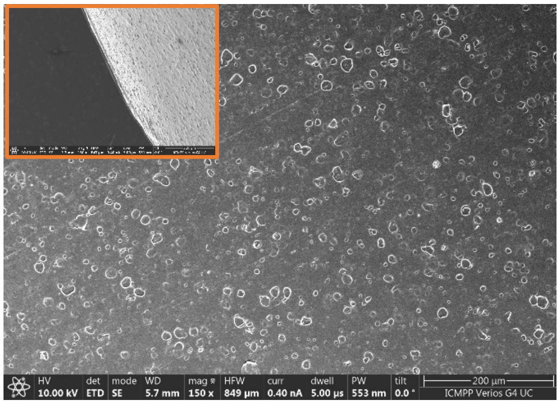

c)

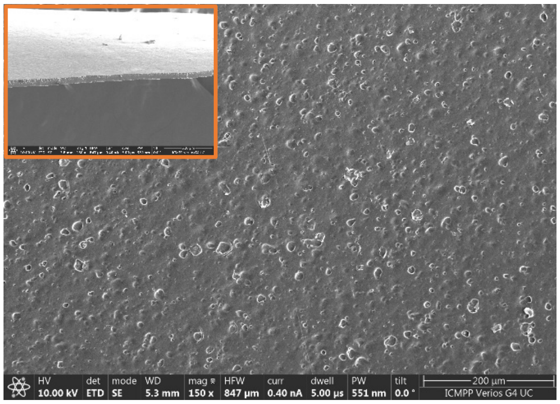

d)

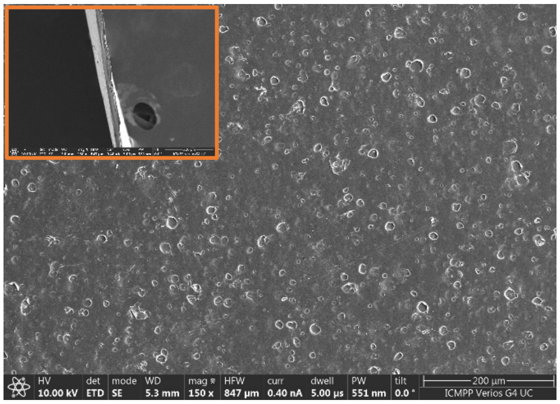

e)

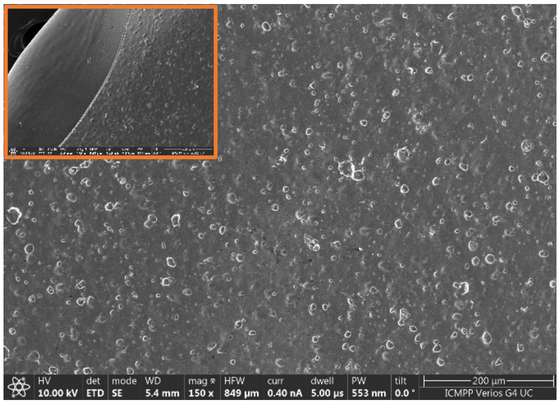

f)

Figure S15. SEM images at a larger scale 200 µm (inset cross section)

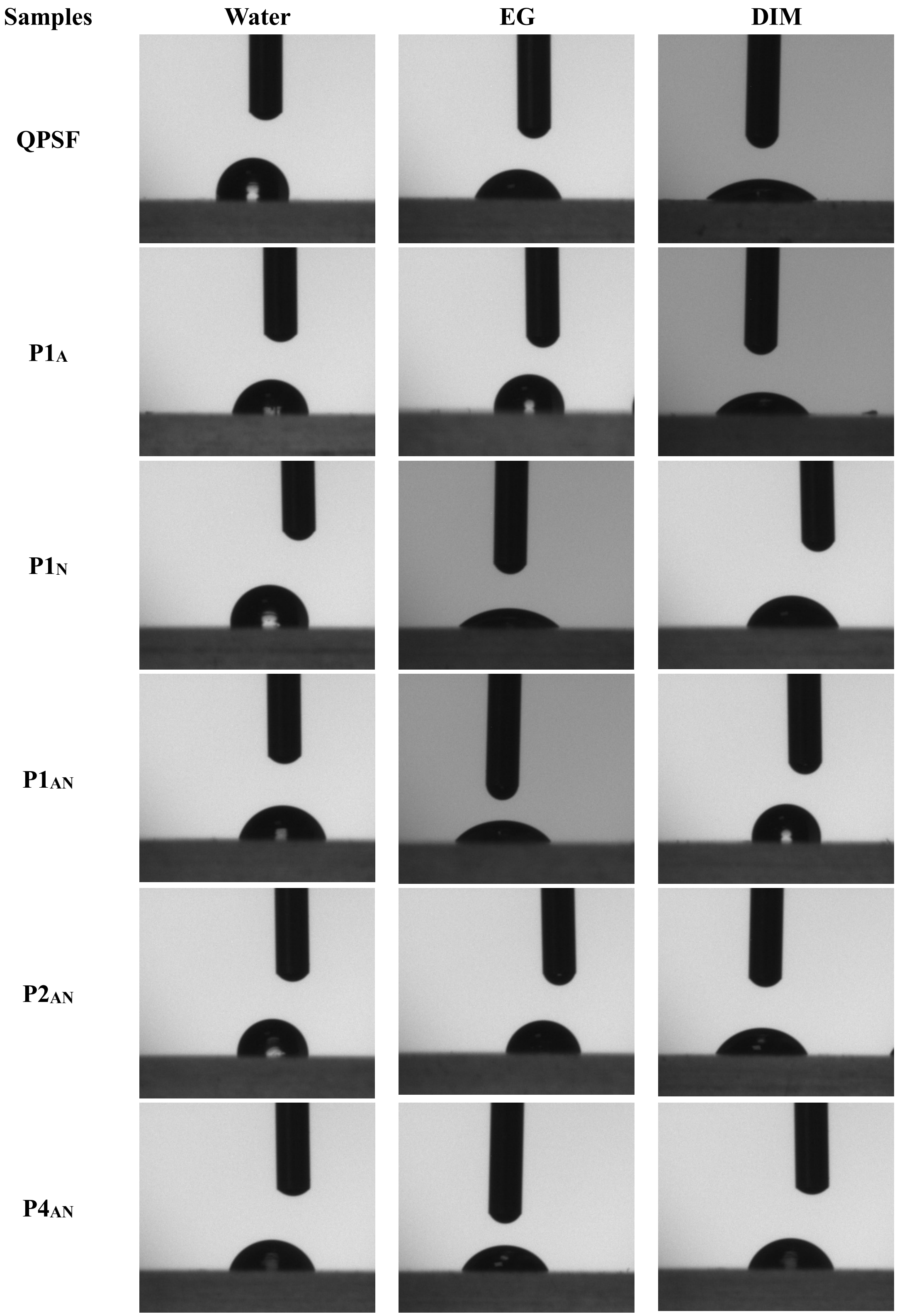

Figure S16. Images of contact angle of water, ethylene glycol (EG) and diiodomethane (DIM) on the surface of the studied samples

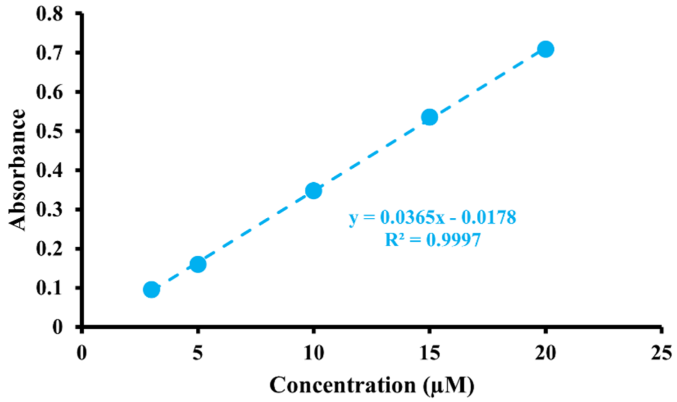

Figure S17. Calibration curve of NFX

As we previously mentioned, the kinetic release of AmB drug from the polymer matrix was not possible due to the low molar concentration of AmB in the sample. For example, in the case of sample P_A, if the entire amount of AmB encapsulated in the polymer matrix was released, the molar concentration of the drug in PBS would be $9.79 \cdot 10^{-6}$ M, and an AmB solution with a concentration of 10^{-6} M in PBS presents an absorbance of 0.05 at 408 nm, a very low value, which will introduce large errors in determining the percentage of drug released (Figure S12). Also, in the case of samples with a higher AmB content ($P2_{AN}$ and $P4_{AN}$), the molar concentration of the drug in PBS would be $2.62 \cdot 10^{-5}$ M and $5.8 \cdot 10^{-5}$ M, respectively, if the entire amount of drug was released. Although the molar concentration of the drug is slightly higher than in the case of samples P_A and $P1_{AN}$, the absorption bands characteristic of the drug does not appear in the UV spectrum of the release medium (Figure S18).

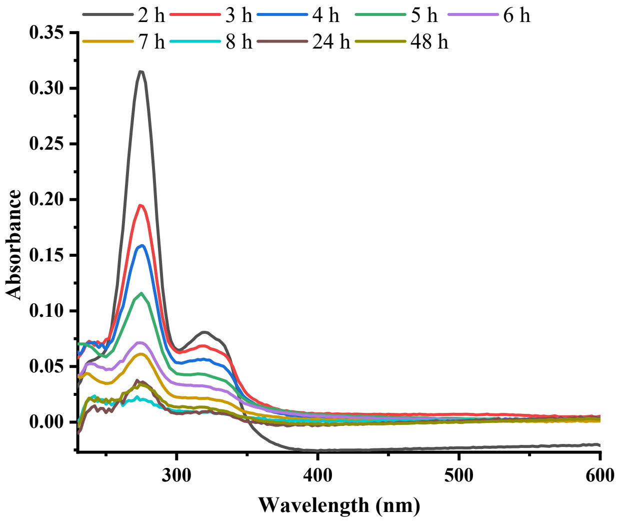

Figure S18. UV spectra of $P2_{AN}$ sample recorded at different periods of time for the drug release kinetics

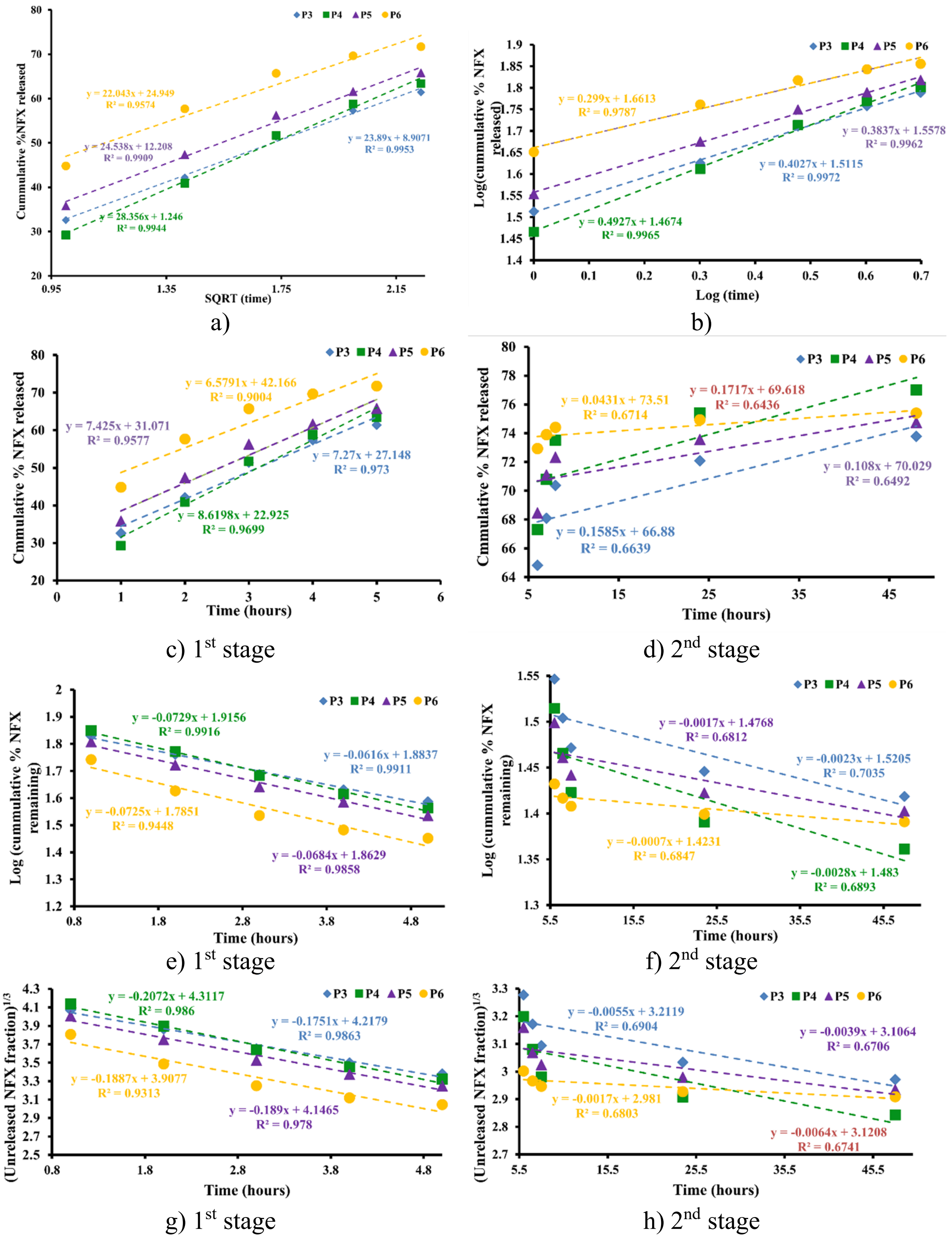

Figure S19. Linear forms of the Higuchi (a), Korsmeyer-Peppas (b), zero-order (c and d), first- order (e and f) and Hixon (g and h) models applied for the release of NFX from the investigates materials for the two release stages

Table S1. EDAX analysis of the samples

Element	QPSF	P_N	P_A	$P1_{AN}$	$P2_{AN}$	$P4_{AN}$
**C**	80.54	80.66	80.44	80.63	80.29	80.01
**O**	10.01	10.53	10.82	10.73	10.4	10.67
**N**	3.54	3.18	2.96	2.92	3.66	3.73
**S**	2.42	2.29	2.39	2.35	2.31	2.25
**Cl**	2.68	2.67	2.62	2.61	2.53	2.53
**F**	-	-	0.27	0.18	0.27	0.25

Table S2. The molar ratio of the components into the investigated samples (+ represent the content of quaternary units and – represent the content of drugs)

Sample	ν_{QPSF}	ν_{NFX}	ν_{AmB}	+/- ratio
**QPSF**	$2.5 \cdot 10^{-4}$	$-$	$-$	$2.5 \cdot 10^{-4}$
P_N	$2.5 \cdot 10^{-4}$	$4.07 \cdot 10^{-6}$	$-$	$2.63 \cdot 10^{-4}/4.07 \cdot 10^{-6}$
P_A	$2.5 \cdot 10^{-4}$	$-$	$1.4 \cdot 10^{-6}$	$2.53 \cdot 10^{-4}/2.24 \cdot 10^{-5}$
$P1_{AN}$	$2.5 \cdot 10^{-4}$	$4.07 \cdot 10^{-6}$	$1.4 \cdot 10^{-6}$	$2.65 \cdot 10^{-4}/2.65 \cdot 10^{-5}$
$P2_{AN}$	$2.5 \cdot 10^{-4}$	$8.14 \cdot 10^{-6}$	$2.8 \cdot 10^{-6}$	$2.78 \cdot 10^{-4}/5.29 \cdot 10^{-5}$
$P4_{AN}$	$2.5 \cdot 10^{-4}$	$16.28 \cdot 10^{-6}$	$5.6 \cdot 10^{-6}$	$3.05 \cdot 10^{-4}/1.06 \cdot 10^{-4}$

3. References

1. Yang, J.; Li, Q.; Oluf, J.; Pan, C.; Cleemann, L.N.; Bjerrum, N.J.; He, R. Phosphoric Acid Doped Imidazolium Polysulfone Membranes for High Temperature Proton Exchange Membrane Fuel Cells. *J Power Sources* **2012**, *205*, 114–121, doi:10.1016/j.jpowsour.2012.01.038.

2. Yang, J.; Wang, J.; Liu, C.; Gao, L.; Xu, Y.; Che, Q.; He, R. Influences of the Structure of Imidazolium Pendants on the Properties of Polysulfone-Based High Temperature Proton Conducting Membranes. **2015**, *493*, 80–87, doi:10.1016/j.memsci.2015.06.010.

3. Liu, Y.; Wang, J. Preparation of Anion Exchange Membrane by Efficient Functionalization of Polysulfone for Electrodialysis. *J. Membr. Sci.* **2020**, *596*, 117591.

4. Avram, E. Polymers with Pendent Functional Groups. VI. A Comparative Study on the Chloromethylation of Linear Polystyrene and Polysulfone with Paraformaldehyde/Me3SiCl. *Polym. Plast. Technol. Eng.* **2001**, *40*, 275–281.
